# Remodelling of Skeletal Muscle During Iron Deficiency Anaemia and Recovery After Intravenous Iron Therapy

**DOI:** 10.1002/jcsm.70415

**Published:** 2026-11-04

**Authors:** Elke Pertler, Sonja A. Wagner, Marlene Panzer, Laura Obholzer, Ylena Sonnenburger, Maria R. Troppmair, Martina Saretto, Benedikt Schaefer, Claudia Manzl, Herbert Oberacher, Bettina Sarg, Klaus Faserl, Stefan Redl, Michael W. Hess, Theresia Telser, Jessica P. Popottnigg, Petra Gronich‐Wondrak, Herbert Tilg, Heinz Zoller

**Affiliations:** ^1^ Christian Doppler Laboratory for Iron and Phosphate Biology, Department of Internal Medicine I Medical University of Innsbruck Innsbruck Austria; ^2^ Department of Internal Medicine I, Gastroenterology, Hepatology and Endocrinology Medical University of Innsbruck Innsbruck Austria; ^3^ Institute of Neuropathology and Neuromolecular Pathology Medical University of Innsbruck Innsbruck Austria; ^4^ Institute of Legal Medicine and Core Facility Metabolomics Medical University of Innsbruck Innsbruck Austria; ^5^ Institute of Medical Biochemistry, Protein Core Facility, Biocenter Medical University of Innsbruck Innsbruck Austria; ^6^ Institute of Neuroanatomy Medical University of Innsbruck Innsbruck Austria; ^7^ Institute of Histology and Embryology Medical University of Innsbruck Innsbruck Austria; ^8^ Institute of Human Genetics Medical University of Innsbruck Innsbruck Austria

**Keywords:** intravenous iron therapy, iron deficiency anaemia, pharmacokinetics, skeletal muscle, skeletal muscle function

## Abstract

**Background:**

The global prevalence of iron deficiency anaemia (IDA) is about 25% and even higher in women of reproductive age and children. IDA can impair skeletal muscle by disrupting oxygen utilization, energy production and contractile function. Haematological effects of intravenous (IV) iron therapy in IDA are well studied, but its impact on muscle and energy metabolism is less clear. We investigated the molecular and functional effects of IDA on skeletal muscle in a mouse model and C2C12 myotubes. The effects of different IV iron formulations on skeletal muscle were compared in iron deficient C2C12 myotubes, in IDA‐mice and patients with IDA.

**Methods:**

Treatment effects of the IV iron formulations ferric carboxymaltose (FCM) and ferric derisomaltose (FDI) were studied in IDA mice by tail vein injection of 2‐mg iron. Analyses were carried out 7 days and 4 weeks post‐treatment. C2C12 myotubes were treated with desferrioxamine, followed by incubation with FCM or FDI before mitochondrial respiration, gene expression and metabolite profiles were analysed. Respiratory muscle function was investigated in adults with IDA treated with FCM or FDI as exploratory endpoint in a randomized clinical trial.

**Results:**

In our mouse model IDA induced a trend towards impaired skeletal muscle function and a shift to anaerobic metabolism. Only treatment with FDI resulted in a significant increase in muscle type I and type IIb fibre cross‐sectional area when compared to IDA. FDI also induced earlier recovery of contractile protein expression and energy metabolism than FCM. Exploratory proteomic and metabolic analyses revealed that treatment with FDI might restore mitochondrial enzymes and reduce lactate accumulation after 7 days. In vitro, iron‐deficient C2C12 myotubes showed reduced mitochondrial respiration and increased glycolysis with partial recovery by both IV iron formulations. Two weeks after treatment of IDA in adults, FDI caused a significantly better improvement in maximal expiratory pressure than FCM (mean pressure difference between treatments 5.06 cmH_2_O, *p* = 0.013). This difference appeared unrelated to post‐treatment hypophosphatemia.

**Conclusions:**

IDA shows a trend towards impaired skeletal muscle function through metabolic and structural remodelling in mice, which is restored with different kinetics by FDI and FCM. These findings are in accord with exploratory findings from a clinical study indicating better respiratory muscle recovery in FDI‐treated than FCM‐treated IDA patients. Cell culture experiments confirm the effects of IDA on impaired metabolism but did not show differential effects of FCM and FDI. Different tissue pharmacokinetics could explain the distinct in vivo response of both formulations.

## 1 Introduction

Iron deficiency anaemia (IDA) affects approximately 25% of the global population, with significantly higher prevalence in females of reproductive age and children [1, 2, 3, 4]. The haematological consequences of IDA with microcytic and hypochromic anaemia as well as thrombocytosis are well established. Iron deficiency also affects tissues with high energy demands, including the brain, heart and muscle. Skeletal muscle function is dependent on sufficient oxygen delivery for energy production by oxidative phosphorylation. Insufficient oxygen supply due to anaemia or reduced oxygen utilization caused by reduced activity of iron‐dependent enzymes can cause fatigue and reduced exercise capacity [5].

Iron plays a critical role in skeletal muscle biology, serving as a cofactor for mitochondrial respiratory chain complexes, enzymes of oxidative metabolism and myoglobin [6, 7]. Iron deficiency not only reduces oxygen transport by causing anaemia but also disrupts energy production, impairs contractile function and induces structural alterations including fibre atrophy and disorganization [8, 9, 10, 11]. Animal and human studies show that intravenous (IV) iron can correct iron deficiency and exercise performance, but direct mechanistic evidence linking iron repletion to the structural and metabolic recovery of iron deficiency in skeletal muscle is missing [12, 13, 14].

Ferric carboxymaltose (FCM) and ferric derisomaltose (FDI) are widely used IV iron formulations with different carbohydrate moieties and distinct pharmacokinetic profiles [15, 16, 17]. Both effectively correct anaemia and restore body iron stores [18, 19]. However, hypophosphatemia is a recognized adverse effect of IV iron therapy, occurring in approximately 47%–75% of patients treated with FCM, whereas FDI treatment shows lower rates of 4%–8% [18, 19, 20]. Studies in athletes with iron deficiency have shown that FCM can improve exercise capacity, but their effect on the structure and function of skeletal muscle is less well characterized [21]. Furthermore, it remains unclear how and when mitochondrial function and muscle architecture are restored after IV iron treatment [22, 23].

To investigate the effects of IDA and its treatment with IV iron, we examined changes in muscle morphology, function and energy metabolism following treatment with FCM or FDI in a mouse model of IDA. The metabolic response of myotubes to iron depletion and repletion with IV irons was further investigated in differentiated C2C12 murine myoblasts. Clinical effects were also studied in an exploratory analysis of respiratory muscle function in a prospective clinical trial comparing the treatment effects of FCM and FDI in patients with IDA.

## 2 Material and Methods

### 2.1 Animal Experiments

All animal experiments were conducted in accordance with the guidelines of the Medical University of Innsbruck and the Austrian Federal Ministry of Science and Research (BMWF‐66.011/0017‐II/3b/2014, protocol numbers 2020–0.554.014 and 2024‐0.534.384). Three‐week‐old C57BL/6J mice from Charles River Laboratories Germany (C57BL/6J) were housed under standard conditions in groups of five per cage at the Innsbruck animal facility. To reduce biological variability, experiments were conducted in female mice. Mice had access to food and water ad libitum and were maintained on a 12‐h light/dark cycle at 20°C–24°C. Animals were separated into four different groups: a healthy control group; an IDA group receiving 0.9% NaCl and two IDA groups subsequently treated IV with 2 mg (100 mg/kg) of either FCM or FDI (Figure 1F). IDA was induced by iron‐deficient diet and repeated phlebotomy before IV iron treatment [24]. IDA was confirmed by reduced haemoglobin, MCV and body weight (Figure S1). Wire hang test was assessed 4 weeks post treatment. Gastrocnemii, solei, plantares and diaphragms were collected one and 4 weeks after IV administration.

**FIGURE 1:**
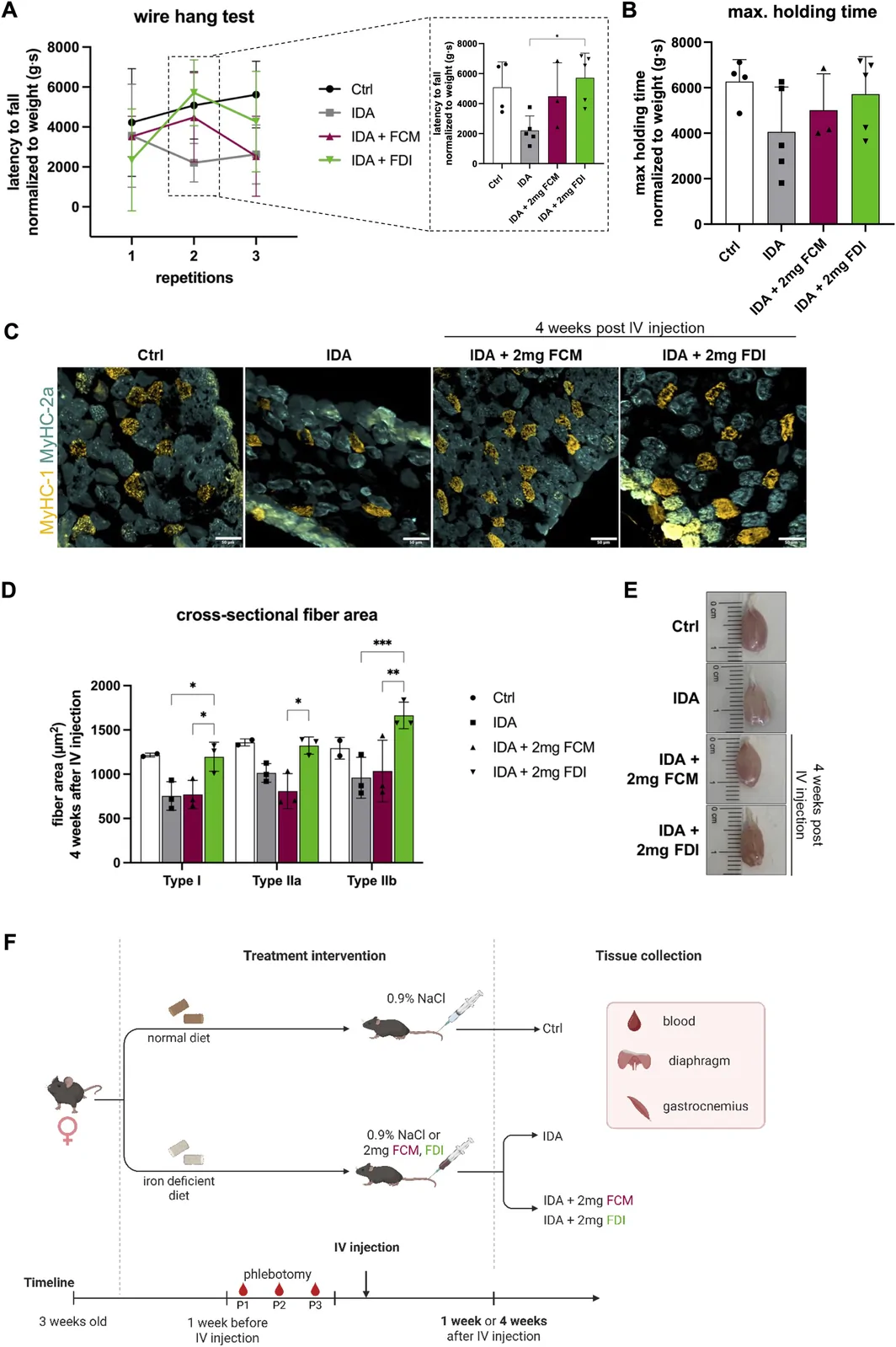
Effects of iron deficiency anaemia and intravenous iron treatment on muscle function and morphology in mice. (A) Latency to fall over three repetitions is shown during the wire hang test for the assessment of the muscle function in untreated control group (Ctrl, *n* = 4), iron deficiency anaemia group (IDA, *n* = 5) and IDA groups treated with ferric carboxymaltose (FCM, *n* = 3) or ferric derisomaltose (FDI, *n* = 5) 4 weeks after treatment. Holding time was recorded up to a maximum duration of 5 min. Wire hang performance was expressed as holding impulse, calculated as latency to fall multiplied by body weight (g·s). The results are shown as mean ± SD Groups were analysed for statistical difference by two‐way and one‐way ANOVA, followed by Tukey's post hoc test. These analyses were exploratory due to the small sample size. (B) Maximum holding among three repetitions during the wire hang test for the assessment of the muscle strength. The latency to fall was normalized to the body weight of the mice. The results represent the mean ± SD. Groups were analysed for statistical difference by one‐way ANOVA, followed by Tukey's post hoc test. These analyses were exploratory due to the small sample size. (C) Representative pictures of the myosin heavy chain (MyHC) staining of the cross sections of diaphragm of each group 4 weeks after the injection. Slow twitch Type I fibres (MyHC‐1) are stained in yellow and fast twitch Type IIa fibres (MyHC‐2a) are stained in blue. Scale bar represents 50 μm. (D) Cross‐sectional fibre area of the fibres Type I, Type IIa, Type IIb of the diaphragm of untreated (*n* = 2) and treated mice (*n* = 3) 4 weeks after the IV injection. The results represent the mean ± SD. IDA, FCM and FDI groups were analysed for statistical difference by two‐way ANOVA. These analyses were exploratory due to the small sample size. (E) Representative pictures of murine skeletal muscles gastrocnemius, soleus and plantaris of healthy mice, IDA mice and IDA mice followed by IV iron treatment. Representative muscles show differences in their colour and size related to their treatment. (F) Schematic representation of the murine model of iron deficiency anaemia (IDA). IDA was induced in 3‐week‐old C57BL/6 mice via an iron‐deficient diet combined with serial phlebotomies (P1–P3). Mice were intravenously (IV) administered either 0.9% saline (NaCl), 2‐mg ferric carboxymaltose (FCM) or ferric derisomaltose (FDI). Tissues and blood were harvested for analysis at 1 and 4 weeks post‐injection. Statistical significance is annotated as follows: *p ≤ 0.05, **p < 0.01 and ***p ≤ 0.001.

### 2.2 Cell Culture Experiments

Murine C2C12 myoblasts were maintained in standard growth medium and differentiated into myotubes under low‐serum conditions for 9 days. To induce iron deficiency, cells were treated on day seven of differentiation with 100 μM desferrioxamine (DFO) for 24 h, followed by another 24‐h incubation with either 100‐μM DFO or with 200 μg/mL of FCM or FDI. Oxygen consumption rate (OCR) was assessed using the Seahorse XF Cell Mito Stress Test Kit (Agilent, #103010‐100) following the manufacturer's protocol.

### 2.3 Muscle Strength Measurements in Patient Cohort

Maximal inspiratory pressure (MIP) and maximal expiratory pressure (MEP) analyses were performed in patients from a previously published randomized trial of FCM versus FDI in adults with iron deficiency anaemia [18]. Briefly, the trials included adult patients with iron‐deficiency anaemia of different etiologies, including abnormal uterine bleeding, gastrointestinal diseases, cancer, bariatric procedures and other conditions leading to significant blood loss, who had documented intolerance or unresponsiveness to oral iron therapy prior to trial enrollment. The majority of enrolled patients had gynaecological causes of iron deficiency anaemia (68.0% in the FDI arm and 69.2% in the FCM arm). Full trial details have been previously reported by Wolf et al. [18]. This analysis is based on data from two prospective cohorts (ClinicalTrials.gov identifiers: NCT03238911 and NCT03237065). In the original Phosphare‐IDA trials, the primary endpoint was the incidence of hypophosphatemia, defined as serum phosphate < 2.0 mg/dL at any time from baseline to day 35. Haematological efficacy outcomes, including changes in haemoglobin, ferritin and transferrin saturation, were secondary endpoints, whereas muscle‐related outcomes, including MIP and MEP, were predefined exploratory endpoints. In the present analysis, the investigational medical product iron isomaltose (IIM) used in the original study was renamed FDI after the study had been completed. Muscle strength measurements were conducted on baseline (FCM, *n* = 113; FDI, *n* = 120), Day 14 (FCM, *n* = 108; FDI, *n* = 110) and Day 35 (FCM, *n* = 110; FDI, *n* = 110). In both treatment groups, patients were additionally stratified according to serum phosphate at follow‐up (≥ 2.0 mg/dL vs. < 2.0 mg/dL). These subgroup analyses were performed to examine whether the observed treatment effects were consistent across phosphate strata and to minimize potential confounding by treatment‐related hypophosphatemia. Descriptive statistics were based on observed cases, and no imputation of missing data was performed. Model‐based estimates were derived from a mixed model for repeated measures including study, treatment, day and phosphate subgroup as factors, relevant interaction terms and baseline value as a covariate.

### 2.4 Statistical Analyses

Data are presented as mean ± SD, unless otherwise stated. For normally distributed parameters, comparisons between more than two groups were performed using one‐way ANOVA followed by Tukey's post hoc test. For analyses involving two independent factors, two‐way ANOVA was performed, followed by an appropriate multiple‐comparisons test as indicated. Non‐normally distributed parameters were analysed using Kruskal–Wallis test followed by Dunn's post hoc test. Two‐group comparisons were performed using unpaired Student's *t* test. For proteomic analyses, Benjamini–Hochberg correction was applied to account for multiple testing, with no proteins reaching statistical significance after adjustment. Therefore, volcano plot, GO enrichment and network analyses were considered exploratory and were based on nominal *p* values combined with an absolute log_2_ fold change threshold of 0.5. A *p* value ≤ 0.05 was considered statistically significant. Unless otherwise stated, all statistical analyses were performed using GraphPad Prism Version 10.1.2 (GraphPad Software, San Diego, CA, USA).

### 2.5 Additional Methods

Detailed descriptions of all methods and additional experimental and analytical procedures are provided in the Supporting Information Methods. Tables [Link], [Link] provide additional methodological details and supporting data.

## 3 Results

### 3.1 Iron Deficiency Anaemia–Induced Atrophy and Impaired Muscle Function Are Partially Corrected by IV Iron Treatment

To assess the impact of IDA and the effect of IV iron treatment on muscle structure and function, we compared untreated control mice (Ctrl), animals with IDA and IV iron‐treated mice 4 weeks after the injection of either FCM or FDI.

The wire hang test showed that IDA and FCM‐treated mice had a shorter latency to fall than control mice or FDI‐treated mice. While iron repleted animals exhibited a gradual increase in holding time on each repetition, IDA mice showed a progressive reduction in the latency to fall, indicating impaired endurance (Figure 1A,B). FDI‐treated mice improved significantly on the second repetition, whereas FCM‐treated mice did not, but mean holding time declined on the third repetition in both IV iron treated groups.

Next, we stained diaphragm tissue samples for the different fibre types to assess effects of IDA and IV iron treatment. This showed a trend towards reduced cross‐sectional area of all fibre types in IDA mice when compared with controls (Figure 1C,D). Treatment with FDI, but not FCM, significantly increased the cross‐sectional area of Types I and IIb fibre types 4 weeks after the injection, but not after 1 week (Figure S2A). At this earlier time point, the cross‐sectional area of oxidative (Type I) fibres correlated significantly with haemoglobin concentrations across all groups (*r* = 0.28; *p* = 0.03, Figure S2B). At 4 weeks post‐treatment, haemoglobin and fibre size further increased in FDI‐treated mice, whereas only haemoglobin, but not fibre size, showed a further increase in FCM‐treated animals (Figure S2C). Muscles were smaller and paler in IDA mice, with a recovery after FCM and FDI (Figure 1E). Collectively, these data demonstrate that IDA causes impaired muscle function and atrophy, which show better recovery after FDI than FCM, despite their comparable effects on haemotologic parameters.

### 3.2 Proteomic Analysis Reveals Impaired Energy Metabolism in IDA and Early Recovery of Contractile Proteins After FDI Treatment

Building on the observed morphological and functional impairments of the skeletal muscle in IDA mice, we next determined the proteomic changes associated with IDA and IV iron treatment. The exploratory results from quantitative proteomic profiling of gastrocnemius muscle in Ctrl, IDA and IDA mice 1 week after IV iron treatment with either FCM or FDI are summarized in Figure 2.

**FIGURE 2:**
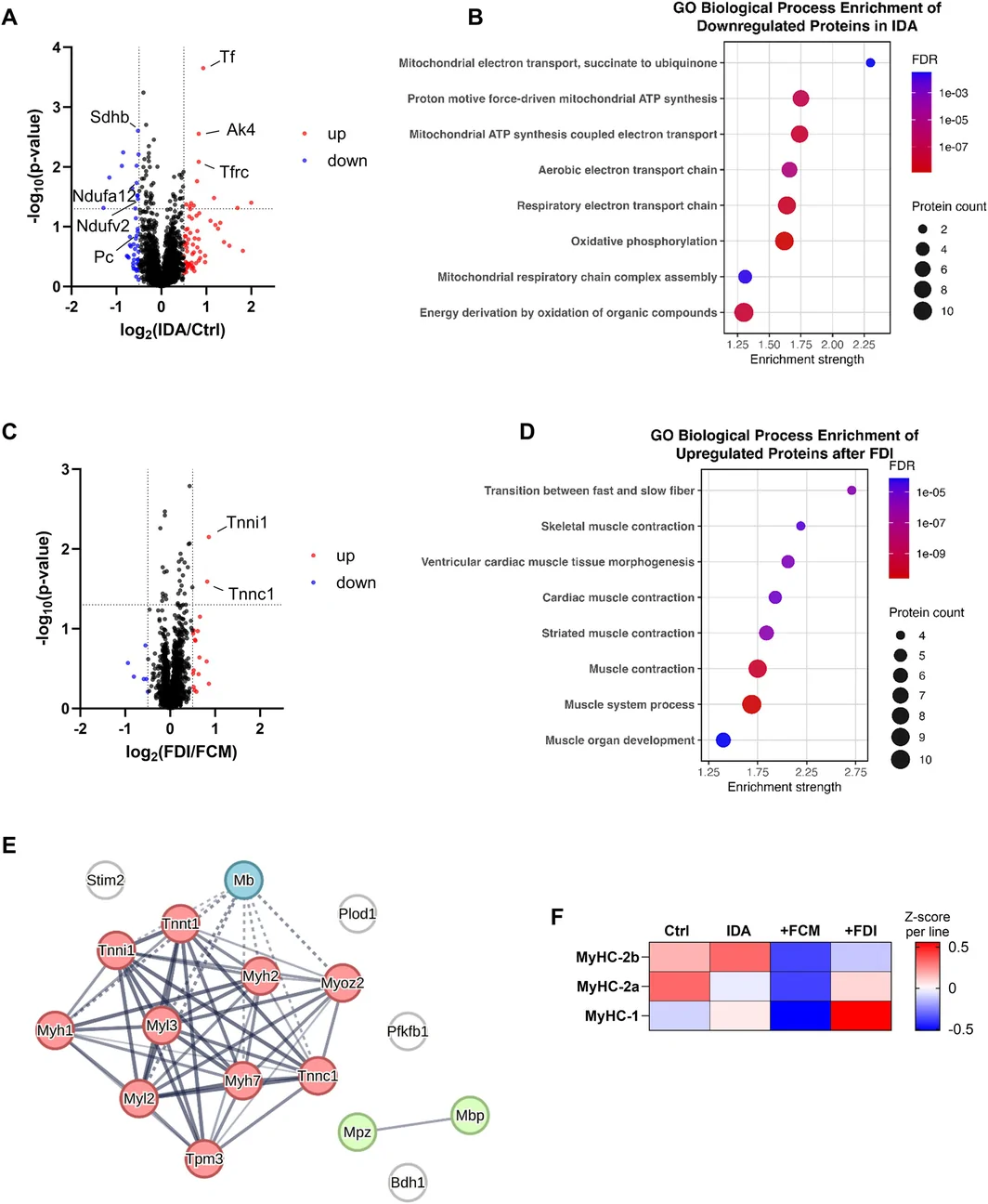
Proteomic profiling of murine skeletal muscle reveals molecular changes in iron deficiency anaemia and after intravenous iron treatment. (A) Analysis of proteomic data of murine skeletal muscle gastrocnemius. Volcano plot is depicted with the fold change of iron deficient anaemic mice (IDA) to mice without treatment (Ctrl) of each protein. The *p* value was calculated by performing a two‐sided Welch's t‐test. The averages of the proteomic expression data of IDA group were compared with the averages of the data for Ctrl group (*n* = 5 per group). Red dots show proteins increased in IDA mice and blue dots show decreased proteins. Iron‐related proteins, including transferrin receptor (Tfrc) and transferrin (Tf) and proteins important for energy metabolism including adenylate kinase 4 (Ak4), Pyruvate carboxylase (Pc), succinate dehydrogenase complex iron sulphur subunit B (Sdhb), NADH dehydrogenase [ubiquinone] 1 alpha subcomplex subunit 12 (Ndufa12) and NADH dehydrogenase [ubiquinone] flavoprotein 2 (Ndufv2) are highlighted in the volcano plot. (B) Gene Ontology (GO) enrichment analysis of the top eight biological processes of downregulated proteins in IDA mice compared to Ctrl (log_2_ fc < −0.5). Pathways are shown on the y‐axis and the enrichment strength on the x‐axis. The colour of the dots represent the FDR and the size of the dots represents the protein count associated with each pathway. (C) Analysis of proteomic data of murine skeletal muscle gastrocnemius. Volcano plot is depicted with the fold change of mice treated with ferric carboxymaltose (FCM) to mice treated with ferric derisomaltose (FDI) 1 week after treatment. The mean of the proteomic expression data for each protein of the FDI group were compared with the mean of the respective protein expression in the FCM group (*n* = 5 per group) and differences in means were tested for significance using a two‐sided Welch's t‐test. Red dots show proteins increased after FDI treatment. Blue dots show decreased proteins. (D) GO enrichment analysis of biological processes of upregulated proteins in FDI treated mice compared to FCM‐treated mice (log_2_ fc > 0.5) Pathways are shown on the y‐axis and enrichment strength on the *x* axis. (E) Clustered protein–protein interaction network analysis for meaningful upregulated proteins in the FDI group compared to FCM. Network nodes correspond to proteins. Lines denote protein–protein associations, line thickness indicates the strength of data support. (F) Protein expression of the different myosin heavy chains (MyHC) characterizing the fibre type, 1 week after IV injection. The heatmap shows the mean *Z*‐score of the proteins for each fibre type of healthy control, IDA and treated IDA mice with either FCM or FDI (*n* = 5 per group). Differential protein abundance was assessed using two‐sided Welch's *t* tests. Benjamini–Hochberg correction was applied to account for multiple testing. No proteins reached statistical significance after adjustment. Therefore, volcano plot, GO enrichment and network analyses were considered exploratory and were based on nominal *p* values combined with an absolute log_2_ fc threshold, as specified for the respective analyses. The network visualization was performed using STRING (v12.0) and the GO enrichment analysis with R‐studios (v2026.01).

Proteomic analysis was restricted to 2920 proteins for which abundance measurements were available across all individual samples. After Benjamini–Hochberg correction for multiple testing, differences in protein abundances were no longer statistically significant. Therefore, subsequent differential abundance and pathway analyses were considered exploratory. Comparison between control and IDA mice revealed a dysregulation of skeletal muscle protein expression (Figure 2A). Notably, iron‐related proteins such as transferrin receptor (Tfrc) and transferrin (Tf) and adenylate kinase 4 (Ak4) were strongly upregulated in IDA. Mitochondrial NADH dehydrogenase subunits (Ndufa12, Ndufv2) and succinate dehydrogenase complex iron sulphur subunit B (Sdhb) were downregulated, suggesting a disruption of oxidative phosphorylation in IDA animals. Gene Ontology (GO) enrichment analysis of downregulated proteins (fc < −0.5, *n* = 43) in IDA mice confirmed impaired mitochondrial function and ATP synthesis, indicating reduced energy metabolism in skeletal muscle of IDA mice (Figure 2B).

When FDI‐treated IDA mice were compared with FCM‐treated IDA mice, distinct protein expression patterns with higher expression of myosin heavy chains and troponins were observed in the FDI group (Figure 2C). GO enrichment of upregulated proteins by FDI (fc > 0.5, *n* = 17) supported the early activation of pathways related to muscle contraction (Figure 2D). Protein–protein interaction network analysis of the proteins upregulated by FDI identified a cluster enriched in muscle contractile proteins (Figure 2E). Consistently, FDI treatment also induced fast recovery of downregulated protein expression of myosin heavy chains in IDA (Figure 2F). These proteomic analyses suggest faster recovery of contractile machinery in FDI‐treated compared to FCM‐treated animals.

### 3.3 Treatment of IDA Causes an Increase in Muscular Iron Content, and Correction of Upregulated Transferrin Receptor mRNA in IDA

Proteomic analyses indicate impaired energy metabolism in IDA mice and signs of early contractile recovery after FDI treatment. To test if these adaptations are associated with changes in muscular iron and iron‐sensing gene expressions, we next quantified iron concentrations in skeletal muscle. Tissue iron concentrations were numerically lower in IDA than in control mice, but this difference did not reach statistical significance (Figure 3A). One week after IV administration of either FCM or FDI, skeletal muscular iron concentrations were significantly higher and remained elevated for 4 weeks post‐injection. Gene expression analysis revealed a significant upregulation of *Tfrc* mRNA in IDA mice, which was completely reversed 1 week after IV iron therapy and remained low at 4 weeks (Figure 3B). Correspondingly, genes related to iron uptake were upregulated in IDA mice, while genes implicated in iron export and storage were downregulated (Figure 3C). Iron–sulphur (Fe–S) cluster binding mitochondrial peptides were downregulated in IDA mice compared to Ctrl and partially recovered following IV iron treatment (Figure 3D).

**FIGURE 3:**
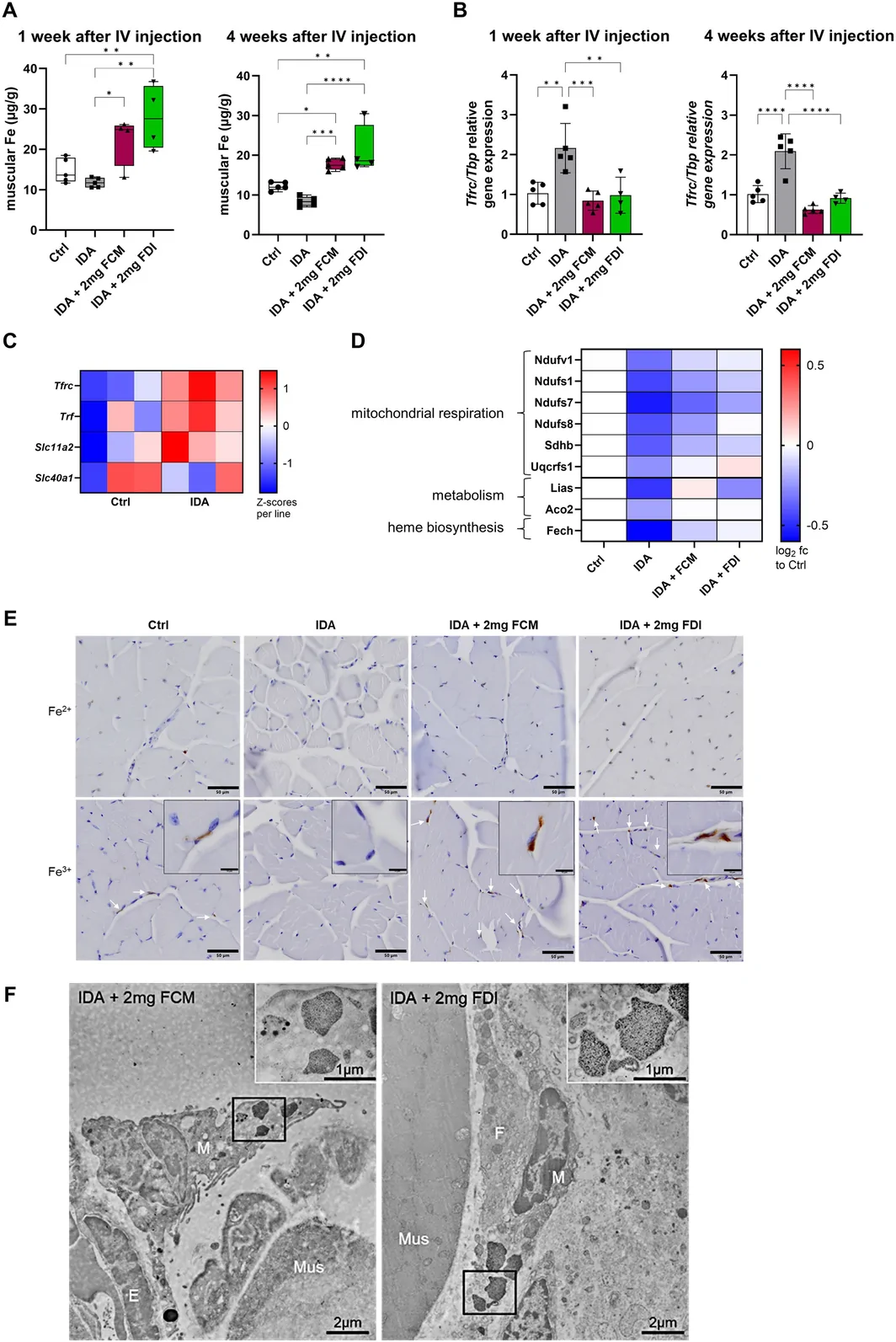
Iron concentration, iron‐associated gene and protein expression and histological iron staining in murine skeletal muscle of control, iron deficient anaemic and iron‐treated mice. (A) Box and whisker plots present the iron concentration of the skeletal muscles gastrocnemius, soleus and plantaris measured by atomic absorption spectrometry and normalized to the weighted tissue. Iron was measured in the untreated control group (Ctrl), the pretreated iron deficient anaemic group (IDA) and different corrected IDA groups with ferric carboxymaltose (FCM) or ferric derisomaltose (FDI) 1 week (Ctrl and IDA: *n* = 5 per group; FDI and FCM: *n* = 4) and 4 weeks (Ctrl, IDA and FCM: *n* = 5 per group; FDI: *n* = 4) after IV injection. The results are presented as mean with minimal and maximal values. Groups were analysed for statistical difference by one‐way ANOVA, followed by Tukey's post hoc test. (B) The relative gene expression of the transferrin receptor *(Tfrc)* normalized to the housekeeping gene TATA‐binding protein *(Tbp)* in the skeletal muscle of all mice (*n* = 5 per group, except FDI *n* = 4) in the different groups 1 and 4 weeks after the IV treatment. The results are shown as mean ± SD Groups were analysed for statistical difference by one‐way ANOVA, followed by Tukey's post hoc test. (C) Heatmap represents transcriptional regulation of representative iron related genes. Genes are presented as *Z*‐scores per line of Ctrl (*n* = 3) and IDA (*n* = 3) mice. Blue shows downregulated genes and red upregulated genes. *Z*‐scores were calculated with fpkm values from RNA‐seq analysis. (D) Adapted expression of mitochondrial proteins binding to iron–sulphur clusters (Fe–S) with their related pathway. The protein expression from proteomic approach are shown as mean of the log_2_ fold change to Ctrl group (*n* = 5 per group). (E) Representative pictures of DAB enhanced ferric and ferrous iron staining (arrows) of the cross‐sections of skeletal muscle gastrocnemius of each group. Iron accumulation 4 weeks after the injection was only found between the fibres. Scale bar represents 50 μm and in zoomed images 10 μm. (F) Representative electron micrographs showing iron loaded endo‐lysosomes in macrophages of skeletal muscle. Scale bar represents 2 μm and in zoomed images 1 μm. muscle tissue (Mus); macrophage (M); fibroblast (F); endothelial cell (E). Statistical significance is annotated as follows: *p ≤ 0.05, **p < 0.01, ***p ≤ 0.001 and ****p ≤ 0.0001.

To determine the cellular distribution of iron after IV treatment, iron was stained by DAB enhanced staining in gastrocnemius muscle. No stainable iron was detected in IDA animals, whereas punctate Fe^3+^‐positive deposits localized to the endomysium were observed 4 weeks after FCM and FDI treatment, without detectable Fe^2+^ (Figure 3E). Stainable iron was restricted to regions between myofibers and no differences were observed between FCM and FDI treatments. Electron microscopy of gastrocnemius muscle from IV iron treated mice confirmed electron‐dense intracellular inclusions in macrophages, identified as iron endo‐lysosomes (Figures 3F and S3).

Taken together, these findings show that treatment of IDA results in elevated tissue iron concentrations, where stainable iron is mainly found in macrophages. Gene expression patterns indicate increased iron uptake and reduced iron storage in IDA, which is reversed by treatment with either IV iron formulation.

### 3.4 FDI Preferentially Reverses IDA‐Induced Shift Towards Anaerobic Glycolysis and Lactate Production

Differences in functional recovery between FCM and FDI treatment may be associated with differences in proteins involved in energy metabolism. We therefore quantified glycolytic and TCA metabolites in skeletal muscle 1 week after IV iron treatment.

Metabolite concentrations in IDA mice indicated a shift from aerobic to anaerobic energy metabolism with elevated intracellular glucose levels that only normalized after FDI but remained high after FCM treatment. The sum of glucose‐/fructose‐6‐phosphate (G/F‐6‐P) remained significantly elevated in IDA relative to all other groups. Pyruvate concentrations decreased following FDI administration and were accompanied by the highest citrate levels. Consistent with reduced oxidative phosphorylation, Pc was strongly downregulated in IDA and remained suppressed after FCM treatment, leading to significant intracellular lactate accumulation (*p* ≤ 0.05), whereas FDI increased Pc expression and reduced lactate. At the protein level, nearly all TCA cycle enzymes were downregulated in IDA relative to controls, with partial recovery 1 week after iron therapy. Iron‐dependent enzymes, including aconitase 2 (Aco2) and multiple succinate dehydrogenase subunits (Sdha, Sdhb), showed pronounced reductions in IDA. In contrast, TCA cycle metabolite concentrations did not differ significantly between Ctrl and IDA. However, treatment‐specific effects emerged beyond citrate, where most other TCA intermediates were lower after FDI compared to FCM (Figure 4).

**FIGURE 4:**
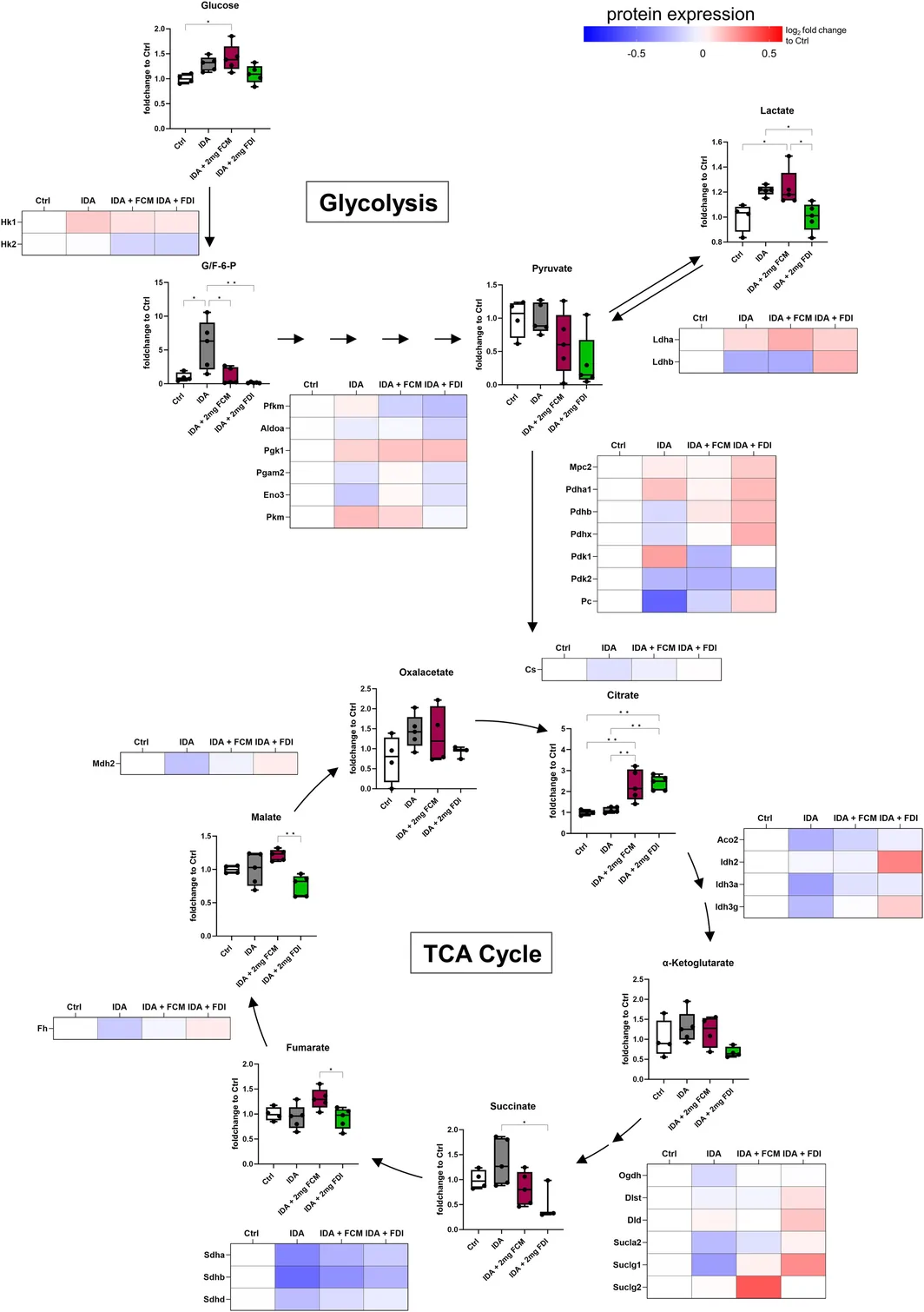
Alterations in glycolysis, TCA cycle metabolites and enzymes in skeletal muscle during iron deficiency anaemia and after iron treatment. Changes of metabolites and enzymes in the tricarboxylic acid (TCA) cycle and the glycolysis are shown between control (Ctrl) mice, iron deficient anaemic (IDA) mice and mice 1 week after treatment with 2 mg of ferric carboxymaltose (FCM) or ferric derisomaltose (FDI). Metabolites are represented as box plots showing the fold change to Ctrl as median with interquartile range; whiskers indicate ± min, max (Ctrl: *n* = 4; IDA, FCM and FDI: *n* = 5). Data points with concentrations below the limit of detection were excluded from the analysis. Heatmaps present the mean log_2_ fold change to Ctrl of related enzymes. Upregulated enzymes are presented in red and downregulated enzymes in blue *(n* = 5 per group). IDA leads to an upregulation of the anaerobic glycolysis, whereas many enzymes of the TCA cycle are downregulated with a recovery after IV iron treatment. Statistical significance is annotated as follows: *p ≤ 0.05 and **p < 0.01.

Collectively, these data show a switch to glycolytic energy production in skeletal muscle of IDA mice, which is mainly reversed by FDI, but less so after FCM.

### 3.5 Metabolic Consequences of Iron Deficiency and the Effects of Iron Repletion in C2C12 Myotubes

Since differences between FCM and FDI treatment in vivo were mainly associated with alterations in energy metabolism, we next employed an in vitro model of iron deficiency to mechanistically dissect these pathways. For this aim, murine myotubes (C2C12) were left untreated, treated with DFO alone or incubated with FCM or FDI after pre‐incubation with DFO.

The analysis of metabolic alterations of DFO‐treated cells compared to Ctrl revealed pronounced accumulation of intermediates in purine and pyrimidine metabolism, glycolysis, fatty acid oxidation and polyamine biosynthesis (Figure 5A). Accumulated metabolites predominantly clustered upstream of iron‐dependent enzymatic steps, indicating a metabolic bottleneck caused by iron deficiency. Conversely, metabolites of the TCA cycle and glycerophospholipid metabolism showed decreased concentrations. Volcano plot analysis confirmed these changes, highlighting metabolites with an absolute log_2_ fold change > 1 and a Benjamini–Hochberg‐adjusted *p* value < 0.05 that indicate a metabolic shift towards anaerobic glycolysis with lactate accumulation in DFO‐induced iron deficiency (Figure 5B). Supplementation with either FCM or FDI partially restored these metabolic alterations with similar effects of both formulations in vitro (Figure 5C,D). Gene expression analysis revealed a significant upregulation of *Ldha* in DFO‐treated cells compared to controls, which was reversed following iron treatment, while elevated extracellular L‐lactate in DFO cultures remained unchanged after iron supplementation (Figure 5E,F).

**FIGURE 5:**
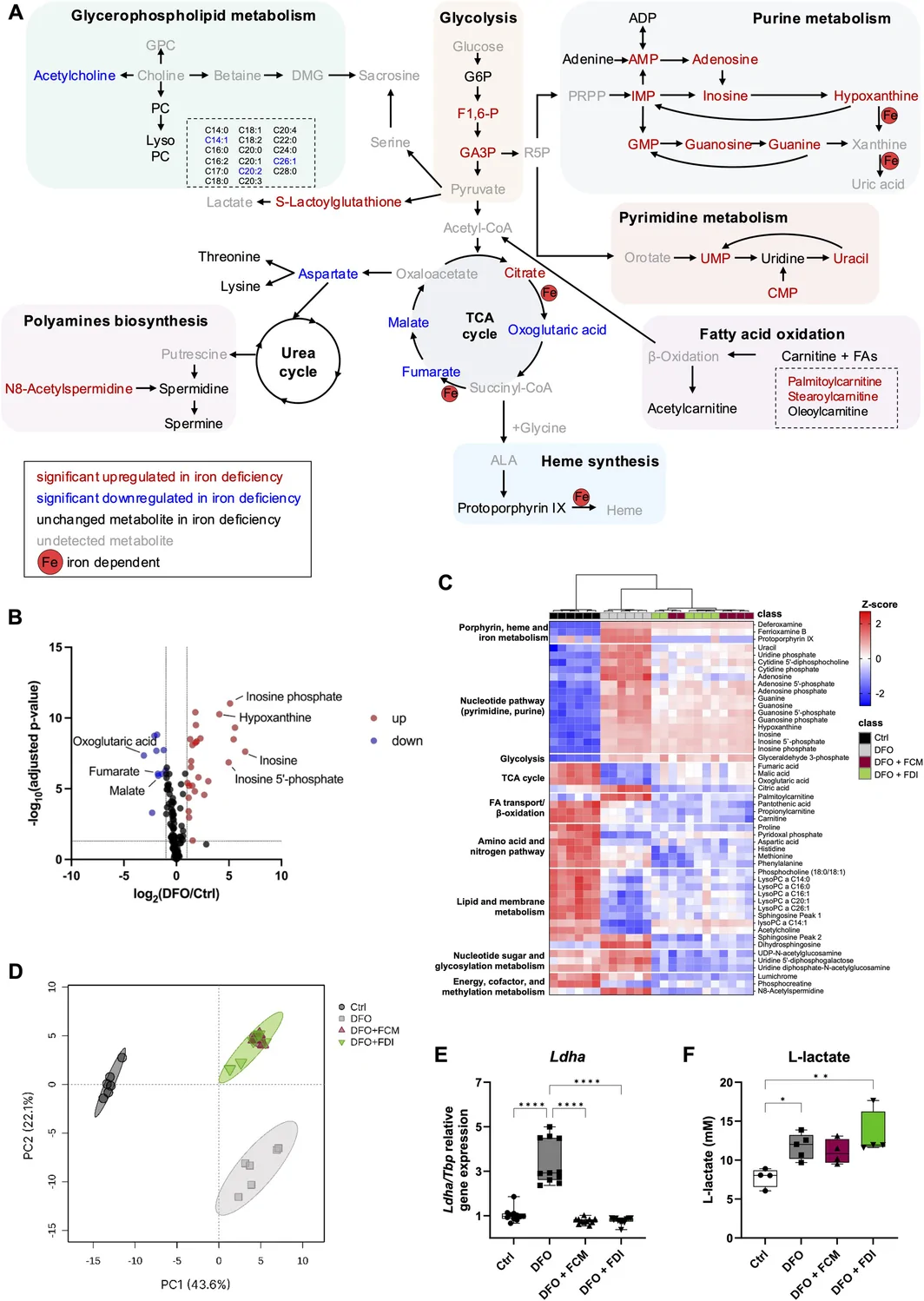
Metabolic alterations in iron deficient C2C12 cells treated with DFO and the adaptation after iron treatment. (A) Schematic representation of central metabolic pathways affected by 100‐μM desferrioxamine (DFO) treatment compared to untreated control cells. Metabolites are colour‐coded according to their regulation status under iron‐deficient conditions: red indicates metabolites significantly upregulated (log_2_ fold change > 1, Benjamini‐Hochberg corrected *p* < 0.05); blue indicates metabolites significantly downregulated (log_2_ fold change < −1, Benjamini–Hochberg corrected *p* < 0.05). Black denotes metabolites that were not altered and grey denoted metabolites that were not detected. Iron dependent steps are highlighted with a red circle. Pathways affected by iron deficiency include glycolysis, TCA cycle, nucleotide pathway, fatty acid oxidation, polyamine biosynthesis and glycerophospholipid metabolism. (B) Volcano plot depicting fold change of DFO‐treated versus control cells (*n* = 6 per group). Dot colour indicates log_2_ fold change (red = upregulate, blue = downregulated). Dotted lines indicate significance thresholds of a Benjamini–Hochberg‐adjusted *p* value of 0.05 and an absolute fold change > 2. (C) Heatmap showing the top 50 metabolites across Ctrl, DFO and cells pretreated with 100‐μM DFO followed by 200 μg/mL of FCM (DFO + FCM) or FDI (DFO + FDI) (*n* = 6 per group). Hierarchical clustering was applied to samples and metabolites were sorted to the belonging pathway. Iron formulations showed no variance in their response. Concentrations were log_10_ transformed and the Z‐scores are displayed per row (red = upregulate, blue = downregulated). (D) Principal component analysis (PCA) plot illustrates sample clustering based on global metabolite profiles. PC1 (43.6%) and PC2 (22.1%) account for the majority of explained variance. The clustering shows a separation of Ctrl and DFO cells along PC1 and PC2, whereas DFO + FCM and DFO + FDI samples cluster together (*n* = 6 per group). Each point represents an individual biological replicate. (E) Gene expression of *lactate dehydrogenase A* (*Ldha*) in C2C12 cells is significantly increased in iron deficient DFO cells compared to control and significantly reduced again after incubation with FCM and FDI (*n* = 11 per group). Data represented as median with interquartile range; whiskers indicate ± min, max Groups were analysed for statistical difference by one‐way ANOVA, followed by Tukey's post hoc test. (F) L‐lactate concentrations in culture supernatant of C2C12 cells show a significant release of lactate induced by DFO treatment (*n* = 5) compared to Ctrl (*n* = 4), without recovery by iron treatment (*n* = 4). Data are shown as median with min and max. Groups were analysed for statistical difference by one‐way ANOVA, followed by Tukey's post hoc test. Statistical significance is annotated as follows: *p ≤ 0.05, **p < 0.01 and ****p ≤ 0.0001. adenosine monophosphate (AMP); 5‐aminolevulinic acid (ALA); cytidine monophosphate (CMP); dimethylglycine (DMG); fatty acid (FA); fructose‐1,6‐bisphosphate (F1,6‐P); glucose‐6‐phosphate (G6P); glyceraldehyde‐3‐phosphate (GA3P); glycerophosphocholine (GPC); guanosine monophosphate (GMP); inosine monophosphate (IMP); lysophosphatidylcholine (LysoPC); phosphatidylcholine (PC); phosphoribosyl pyrophosphate (PRPP); ribose‐5‐phosphate (R5P); uridine monophosphate (UMP).

Collectively, DFO‐induced iron deficiency in cell culture leads to coordinated remodelling of one‐carbon, nucleotide, amino acid and lipid metabolism, indicating a metabolic shift towards anaerobic glycolysis with increased lactate production and impaired fatty acid oxidation. Iron repletion partially restores metabolic profiles, without significant differences between both formulations.

### 3.6 Murine Myotubes Respond to Iron Deficiency With Reduced Oxygen Consumption and Increased Transferrin Receptor mRNA, Which Is Equally Corrected by FCM and FDI

Given that DFO‐induced iron deficiency profoundly remodels central metabolic pathways and shifts energy production towards anaerobic glycolysis, we next assessed whether these metabolic alterations translate into functional impairment of mitochondrial respiration. Seahorse respirometry showed that iron chelation significantly (*p* ≤ 0.0001) reduced basal and maximal oxygen consumption rates (OCRs) when compared to control, indicating impaired mitochondrial function. FCM and FDI led to significant recovery after 24 h, without apparent differences between the formulations (Figure 6A). When myotubes were stressed following treatment with oligomycin and FCCP (carbonyl cyanide‐4‐[trifluoromethoxy] phenylhydrazone), DFO‐treated myotubes exhibited increased glycolytic reliance, whereas FCM‐ and FDI‐treated cells displayed significantly improved, though still incomplete, oxidative adaptability relative to controls (Figure 6B). Immunoblotting revealed reduced expression of oxidative phosphorylation (OXPHOS) complexes in DFO treated cells with recovery after iron treatment, while mitochondrial copy number showed no significant differences after DFO treatment (Figure 6C,D). At the transcriptional level, iron deficiency led to significant (*p* ≤ 0.0001) increase of *Tfrc* mRNA, which returned to baseline following treatment with either iron compound. Direct iron uptake into C2C12 myotubes was confirmed by electron microscopy and FerroOrange staining (Figures 6 and S4). In contrast, mRNA expression of *Dmt1* did not change in response to DFO‐exposure alone or subsequent FCM or FDI treatment. *Zip14* mRNA levels significantly decreased (*p* ≤ 0.01) after DFO, without changing after treatment with either IV iron formulation (Figure 6E).

**FIGURE 6:**
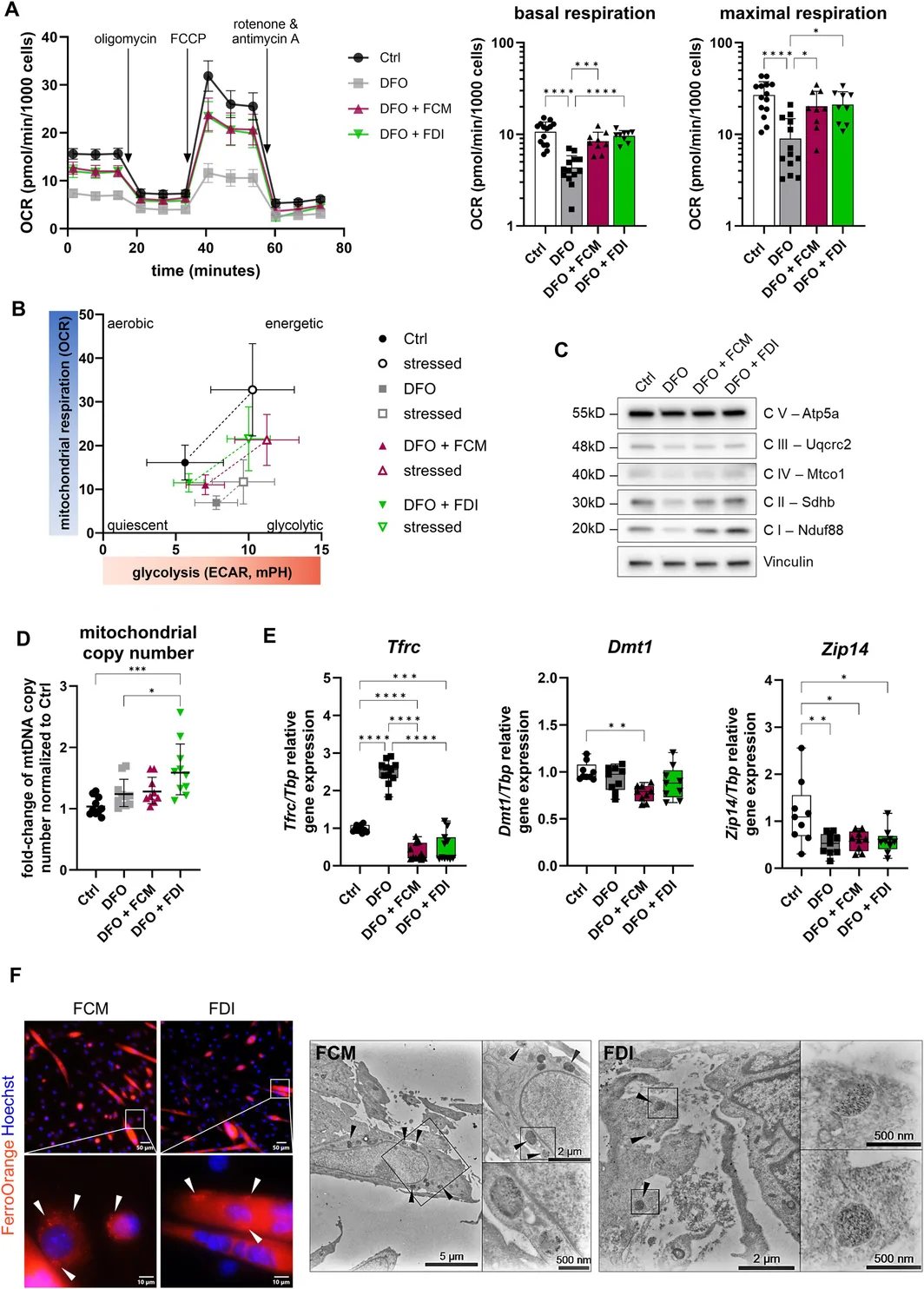
Effects of iron deficiency and iron repletion on mitochondrial respiration and iron transporter expression in C2C12 myotubes. (A) Respirometry traces and respiratory rates from C2C12 myotubes differentiated for 9 days (*n* = 14) and pre‐treated with 100‐μM desferrioxamine (DFO, *n* = 13) for 24 h before cells were incubated for 24 h with either 100 μM DFO or in the presence of 200‐μg/mL ferric carboxymaltose (FCM, *n* = 9) or ferric derisomaltose (FDI, *n* = 9) were added for 24 h. Oxygen consumption rate (OCR) over time was measured with the Seahorse Analyzer XF from Agilent and results normalized to the cell number. Measured OCR at basal respiration and at maximal respiration following the addition of oligomycin and FCCP (carbonyl cyanide‐4‐(trifluoromethoxy) phenylhydrazone) of the untreated control group and treated groups with DFO followed by FCM or FDI. Data are presented as mean ± SD. Groups were analysed for statistical difference by one‐way ANOVA, followed by Tukey's post hoc test. (B) Energy map of the metabolic potential showing the correlation between OCR and extracellular acidification rate (ECAR) of cells at the baseline respiration (filled symbol) and in the stressed state (empty symbol) following the addition of oligomycin and FCCP. Data shown as mean ± SD of all groups. (C) Protein expression of oxidative phosphorylation complexes in treated C2C12 cells. Representative immunoblots for the Complexes I (C I, Ndufb8), II (C II, Sdhb), III (C III, Uqcrc2) and V (C V, Atp5a) with the loading control (Vinculin) of Ctrl, DFO and IV iron treated cells showing comparable protein concentrations in the different treatment groups for all four complexes. (D) Fold change of the copy number of mitochondrial DNA (mtDNA) to nuclear DNA (nucDNA) of Ctrl (*n* = 10), DFO treated (*n* = 10) and pretreated with DFO followed by FCM (*n* = 9) or FDI (*n* = 10) normalized to Ctrl. The mtDNA represents the ratio of *Rpp30* (nucDNA) to *CoxI* (mtDNA). Data are shown as mean ± SD. Groups were analysed for statistical difference by one‐way ANOVA, followed by Tukey's post hoc test. (E) Box and whisker plot presents relative gene expression of the transferrin receptor (*Tfrc*) (*n* = 11)*,* divalent metal transporter 1 (*Dmt1*)*,* Zrt‐ and Irt‐like protein 14 (*Zip14*) in differentiated untreated C2C12 cells and in cells treated with 100 μM DFO followed by 200 μg/mL of FCM or FDI as indicated (*n* = 9 per group). The results are shown with minimal and maximal values of each group. Groups were analysed for statistical difference by one‐way ANOVA, followed by Tukey's post hoc test. (F) Representative images of FerroOrange staining in FCM and FDI exposed C2C12 myotubes. Labile iron is represented in orange and shows a punctate intracellular staining of FCM and FDI‐treated C2C12 cells in addition to the diffuse intracellular staining. Nuclei are stained with Hoechst shown in blue. Arrow heads indicate iron vesicles. Scale bar represents 50 μm and in the zoomed images 10 μm. Representative electron microscopy images of C2C12 myotubes incubated with FCM or FDI. Arrow heads show electron dense endo‐lysosomes harbouring iron in the myotubes. Statistical significance is annotated as follows: *p ≤ 0.05, **p ≤ 0.01, ***p ≤ 0.001 and ****p ≤ 0.0001.

These findings demonstrate a coordinated bioenergetic and transcriptional response to iron depletion in skeletal muscle cells, which improves after FCM and FDI treatment in vitro.

### 3.7 FDI Improves Respiratory Muscle Function in Adults With Iron Deficiency Anaemia

Based on the findings that skeletal muscle function showed a better improvement after FDI than FCM in our IDA mouse model, we next analysed respiratory muscle function in IDA patients treated with either FCM or FDI, which had been collected as exploratory endpoint in the Phosphare‐IDA studies [18]. For this analysis, maximal expiratory (MEP) and inspiratory pressures (MIP) were assessed as surrogates of respiratory muscle function. When muscle function was compared between treatment arms before, 14 and 35 days after treatment with either FDI or FCM, patients treated with FDI showed a significantly stronger increase in MEP than FCM‐treated patients. This increase from baseline was already present 14 days after FDI treatment and was maintained through Day 35 (Figure 7A). Patients treated with FCM showed no significant improvement over baseline during the same period. This difference was less pronounced when MIP measurements were compared between FDI‐ and FCM‐treated patients (Figure 7B). As hypophosphatemia occurred in 74% of patients treated with FCM and in only 8% of patients treated with FDI, the differential effects of FDI and FCM on muscle function were also grouped by post‐treatment phosphate concentrations. The fact that few patients developed hypophosphatemia after FDI limits the statistical power of this analysis, yet no association between hypophosphatemia and the degree of recovery of respiratory muscle function was found (Figure 7C,D).

**FIGURE 7:**
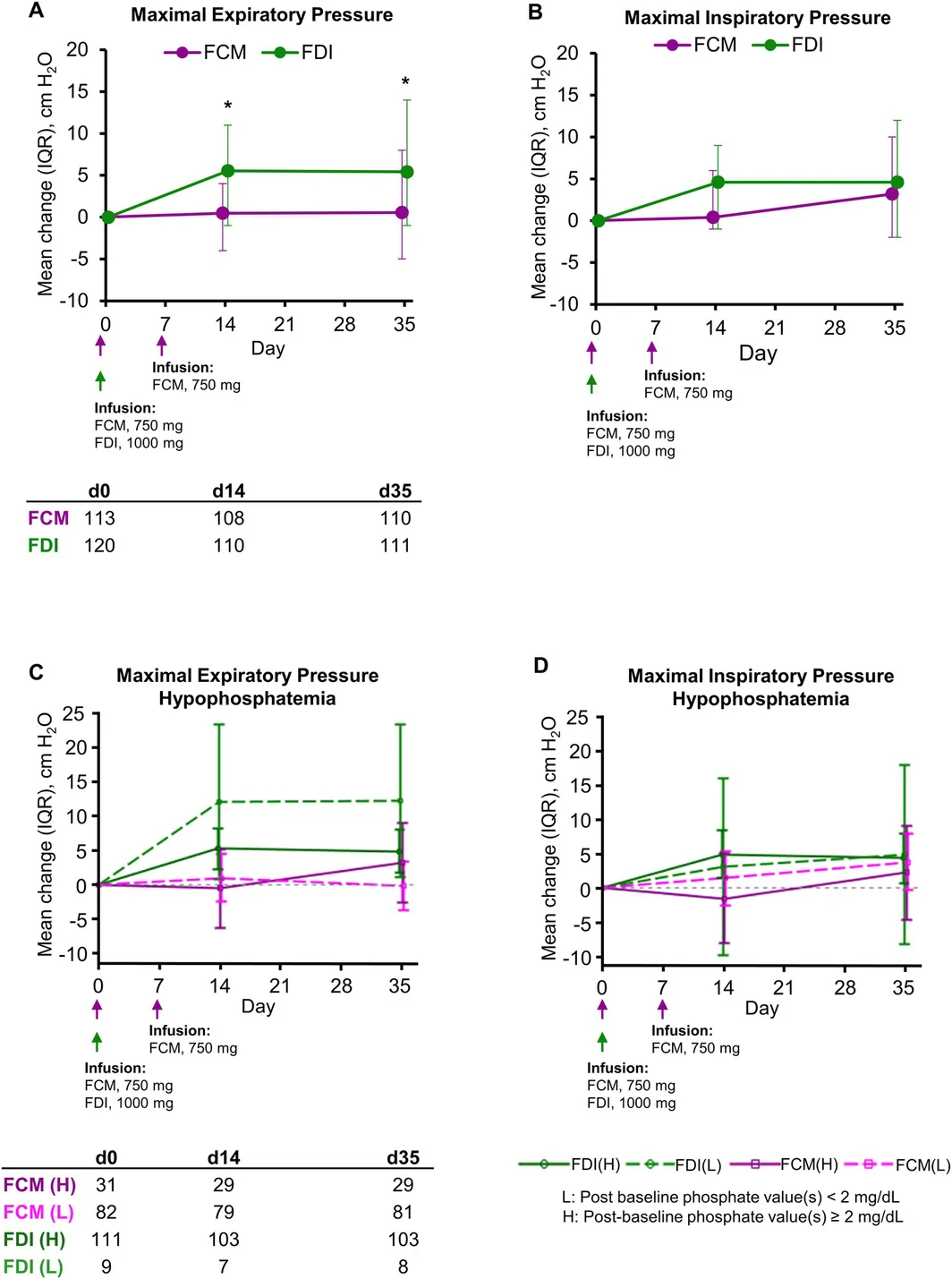
Secondary analysis of muscle function in the cohort of Phosphare‐IDA Trial [18]. (A) Maximal expiratory pressure (MEP) and (B) maximal inspiratory pressure (MIP) of iron deficient anaemic patients treated with either 1000‐mg ferric derisomaltose (FDI) on Day 0 or two times with 750‐mg ferric carboxymaltose (FCM) on Day 0 and Day 7. MIP and MEP were assessed over time on Day 0, 14 and Day 35 after the first IV injection. Data are shown as the absolute mean change. (C) MIP and (D) MEP of patients grouped for FDI and FCM injection and the phosphate status with phosphate ≥ 2 mg/dL (H) and < 2 mg/dL (L) over time on Day 0, 14 and 35 after the first IV injection. Statistical differences between groups were assessed by Restricted Maximum Likelihood (REML)‐based Mixed Models for Repeated Measures (MMRM) approach. Data are shown as absolute mean changes to baseline with CI (5%, 95%). Statistical significance is annotated as follows: *p ≤ 0.05.

These findings suggest that in adults with IDA, FDI treatment can be associated with a more pronounced and more rapid improvement of MEP than FCM treatment, which is consistent with the findings observed in our mouse model.

## 4 Discussion

In mice, IDA caused fibre atrophy, a trend to impaired contractile function and a shift to anaerobic energy metabolism. Treatment with IV iron improved functional, morphological and molecular changes in iron‐deficient muscle. In cultured murine myotubes, FCM and FDI had similar effects on restoring cellular energy metabolism after DFO treatment. Respiratory muscle function in humans with IDA appeared to improve faster after FDI than after FCM treatment.

Earlier studies in humans and animals have shown that iron deficiency can cause impaired energy metabolism in skeletal muscle [11, 25]. Our data reveal metabolic remodelling in IDA uncovering a shift from oxidative phosphorylation towards anaerobic glycolysis, reflected by intracellular lactate accumulation. The observation that several TCA cycle intermediates were reduced and the finding that specific TCA cycle enzymes were higher after FCM than after FDI could be interpreted as increased TCA turnover. Alternative causes for these findings include treatment‐specific differences in anaplerotic and cataplerotic reactions during metabolic recovery. Since metabolite levels do not directly indicate pathway flux, interpretation remains speculative. Similarly, higher glucose abundance observed after FCM in the context of increased lactate concentrations may suggest altered glucose handling in skeletal muscle but cannot be interpreted as beneficial or detrimental without direct assessment of glucose uptake, glycogen content or glycolytic flux [26].

Not only biochemically but also functionally FDI and FCM had distinct properties, where FDI treatment of IDA‐mice led to a faster muscular recovery. These findings were supported by an exploratory analysis of muscle function in humans, where recovery of respiratory muscle function parameter MEP appeared more rapid and pronounced after FDI treatment, despite higher iron doses and greater haemoglobin response after FCM [18].

Prior work indicates that skeletal muscle responds to changes in iron availability through cell‐autonomous and systemic mechanisms, including inter‐organ cross‐talk, ferritinophagy‐driven remodelling of myogenic cells and macrophage‐mediated local iron handling [22, 27, 28]. Accordingly, skeletal muscle of IDA mice had significantly increased *Tfrc* expression, but only a trend towards lower total iron content, suggesting direct iron sensing in muscle. These findings are in accord with recent data showing that even minor changes in cellular iron concentrations can have gross effects on *Tfrc* expression [29]. In contrast, increased bulk muscle concentrations after IV iron therapy do not necessarily indicate repletion of biologically available iron in myofibers because IV iron led to sustained iron accumulation in interstitial macrophages up to 4 weeks. *Tfrc* mRNA levels normalized as early as 1 week after treatment, when metabolic homeostasis was not fully restored. Based on these observations, we hypothesize that effective muscular adaptation in vivo may involve the gradual transfer of macrophage iron to myofibers. However, such macrophage‐to‐myofiber iron transfer was not directly demonstrated in our study.

FDI and FCM can either release trace amounts of labile iron directly into plasma or undergo macrophage uptake with subsequent lysosomal degradation of the carbohydrate shell [16, 30, 31]. In addition to differences in iron release, FCM and FDI can also undergo differential biodistribution into different tissues, which is consistent with our findings of distinct muscular iron bioavailability [16]. Despite comparable haematological responses in our IDA‐mice, FDI consistently produced faster metabolic and functional recovery, which is consistent with data showing that lysosomal iron release from FCM‐treated cells is slower than from other iron formulations [32]. This discrepancy could further reflect differences between both iron formulations in iron‐release kinetics and tissue pharmacokinetics as discussed above.

Recent work has also shown that FCM and FDI differ in their effects on phosphate homeostasis during correction of IDA despite comparable haematological efficacy [33]. In our mouse model, plasma phosphate was only measured 1 week after IV iron treatment and did not show lower phosphate in FCM treated animals, which renders phosphate unlikely driving the observed muscle phenotype [24]. Our findings support a combined mechanism involving systemic anaemia with reduced oxygen transport together with compartment‐specific or functional iron limitation within skeletal muscle that is not fully captured by bulk tissue iron measurements.

Findings in cell culture showed that FCM and FDI equally normalized increased *Tfrc* mRNA expression and energy metabolism after DFO treatment, although broader cellular stress effects of DFO cannot be fully excluded. This indicates that in vitro cells can directly access iron from FCM and FDI circumventing effects caused by differences in tissue pharmacokinetics. Electron micrographs from C2C12 myotubes showed in FCM‐ and FDI‐treated cells endo‐lysosomal organelles filled with electron‐dense particles representing iron‐conjugates, which was confirmed by FerroOrange staining. This indicates that C2C12 myotubes can internalize FCM and FDI complexes in the absence of macrophages. In contrast, electron microscopy from murine muscle fibres did not show such inclusions under the conditions examined. Those endomysial iron accumulations in macrophages observed after IV iron treatment likely reflect local iron sequestration and buffering by interstitial macrophages. While such cells may serve as local iron reservoirs and could contribute to subsequent redistribution to myofibers, our data do not directly demonstrate macrophage‐to‐myofiber iron transfer.

Previous studies have shown that iron deficiency impairs nucleotide synthesis by reduced activity of iron‐dependent enzymes, including ribonucleotide reductase [34, 35, 36, 37]. Our metabolomic analysis of in vitro experiments confirmed impaired nucleotide biosynthesis, showing that DFO caused the strongest increase in inosine phosphate and hypoxanthine. Taken together, these findings highlight that iron deficiency affects cellular metabolism beyond energy production and oxidative phosphorylation.

Limitations of the present study include that functional performance in the wire hang test cannot be attributed to a single physiological component. The wire hang test reflects a combination of muscle strength, neuromuscular function, motivation, coordination and fatigue resistance. This limitation should be considered when interpreting the in vivo functional data, which were complemented by contractile, morphological and molecular analyses of skeletal muscle. Another limitation of the present study is the fact that molecular changes in skeletal muscle were only analysed in animals with severe IDA and cells treated with DFO. Recent data suggest that reduced expression of respiratory chain proteins is also present in humans, but comprehensive proteomic and metabolomics analyses in humans are lacking. A further limitation is the relatively higher IV iron dose used in mice compared with humans on a body weight basis. Still, the translational findings presented in this study suggest that in humans with IDA, FDI could more rapidly improve respiratory muscle function than FCM despite higher FCM dosing, but the clinical relevance of these findings depends on the clinical setting. Respiratory muscle function is of relevance in perioperative medicine. To put the observed effect size of ~5 cmH_2_O higher MEP into context, one study showed that MEP was an independent predictor of outcome with each cmH_2_O translating into 4% lower odds of extubation failure [38]. Although the Phosphare‐IDA study was not conducted in intubated and ventilated patients, the exploratory findings presented here could have potential implications in patients treated with IV iron preoperatively.

In conclusion, our findings demonstrate that IDA can impair skeletal muscle function through coordinated metabolic reprogramming affecting mitochondrial and nucleotide pathways. Correction of IDA with FDI results in faster functional recovery than with FCM, despite equal hematologic response kinetics, whereas C2C12 cells without associated macrophages showed no differences between formulations. These findings suggest that differences in formulations and potentially distinct tissue pharmacokinetics could explain the observed differences in functional outcome in vivo. Our study results can help optimize treatment for IDA and highlight how IV iron treatment can improve skeletal muscle health and function.

## Ethics Statement

The original trials (ClinicalTrials.gov identifiers: NCT03238911 and NCT03237065) were conducted in accordance with the principles of the Declaration of Helsinki and Good Clinical Practice and were approved by a central institutional review board (Western Institutional Review Board, Puyallup, WA, USA). All participants provided written informed consent prior to enrolment. The authors had access only to aggregated results and graphical data provided by Pharmacosmos and no individual patient‐level data were accessed for this analysis.

All mouse experiments were approved by the Medical University of Innsbruck and the Austrian Federal Ministry of Science and Research (BMWF‐66.011/0017‐II/3b/2014) and conducted in accordance with national and institutional guidelines for the care and use of laboratory animals.

## Conflicts of Interest

E.P. reports receiving support for travel to conferences from Pharmacosmos. S.A.W. reports receiving support for travel to conferences and advisory board/consulting fees from Pharmacosmos. M.P. reports receiving support for travel to conferences from Gilead and Pharmacosmos. L.O. has no potential conflict of interest to report. Y.S. has no potential conflict of interest to report. M.R.T. reports receiving support for travel to conferences from Gilead and Abbvie. M.S. reports receiving support for travel to conferences from Pharmacosmos. B.Sch. reports receiving honoraria for lecturing from Pharmacosmos and Astro Pharma and grant support from Astro Pharma. C.M. has no potential conflict of interest to report. H.O. has no potential conflict of interest to report. B.Sa. has no potential conflict of interest to report. K.F. has no potential conflict of interest to report. S.R. has no potential conflict of interest to report. M.W.H. has no potential conflict of interest to report. T.T. has no potential conflict of interest to report. J.P.P. has no potential conflict of interest to report. P.G.‐W. has no potential conflict of interest to report. H.Ti. has no potential conflict of interest to report. H.Z. reports receiving advisory board/consulting fees from Pharmacosmos, Pierre Fabre, Falk, Vifor and Novo Nordisk. H.Z. reports grant support paid to the Medical University of Innsbruck by Vifor and grant support paid to the Christian Doppler Society by Pharmacosmos. H.Z. has received honoraria for lecturing from Pharmacosmos, Falk, Roche and Khedrion Pharma and support for travel to conferences by Roche and Pharmacosmos.

## Supporting information


**Data S1:** Supporting Information.

**Figure S1:** Characterization of the murine model of iron deficiency anaemia
**Figure S2:** Cross‐sectional diaphragm fibre area and its correlation with haemoglobin concentration in mice
**Figure S3:** Electron energy loss spectroscopy (EELS) in skeletal muscle of iron treated mice
**Figure S4:** FerroOrange fluorescence staining of C2C12 myotubes

**Table S1:** ssniff composition of murine diet

**Table S2:** Tissue allocation of all mouse experiments

**Table S3:** Protein abundance matrix.
**Table S4:** Differential protein abundance analysis IDA to Ctrl.
**Table S5:** Differential protein abundance analysis FDI to FCM.
**Table S6:** GO Biological Processes of downregulated proteins (fc < −0.5) in IDA to Ctrl.
**Table S7:** GO Biological Processes of upregulated proteins (fc > 0.5) in FDI to FCM.

**Table S8:** Results of the Quantitative analysis of sugars, phosphosugars and short‐chain carboxylic acids in homogenized tissue samples

**Table S9:** Reference compounds used for preparing quality control samples of metabolomic analysis.
**Table S10:** List of putatively annotated compounds in positive mode data (metabolomic analysis)
**Table S11:** List of putatively annotated compounds in negative mode data (metabolomic analysis)
**Table S12:** List of putatively annotated compounds after normalization to internal reference standards
**Table S13:** Statistics of volcano blot DFO to Ctrl (metabolomic analysis).
**Table S14:** Z‐scores of top 50 putatively annotated compounds for pathway analysis.

**Table S15:** Analysis of Absolute Change in Respiratory Muscles Strength—Maximal Expiratory Pressure (cm H2O) from Baseline to Day 14 and 35—ITT Analysis Set
**Table S16:** Analysis of Absolute Change in Respiratory Muscles Strength—Maximal Inspiratory Pressure (cm H2O) from Baseline to Day 14 and 35—ITT Analysis Set
**Table S17:** Mean change (95% CI) in respiratory muscle strengths from baseline to day 35 by phosphate subgroup (IDA‐04/05) Maximal Expiratory Pressure (cm H2O)
**Table S18:** Mean change (95% CI) in respiratory muscle strengths from baseline to day 35 by phosphate subgroup (IDA‐04/05) Maximal Inspiratory Pressure (cm H2O)

## Data Availability

The RNA sequencing data generated in this study are openly available in the Gene Expression Omnibus (GEO) database under accession number GSE314924. The mass spectrometry proteomics data have been deposited to the ProteomeXchange Consortium via the PRIDE [39] partner repository with the dataset Identifier PXD082925. The metabolomics data supporting the findings of this work are provided in the Supporting Information of this article.
