# Supplementary material for: Remodelling of Skeletal Muscle During Iron Deficiency Anaemia and Recovery After Intravenous Iron Therapy
Source: J Cachexia Sarcopenia Muscle. 2026 Nov 4;17(6):e70415. doi: 10.1002/jcsm.70415 (PMC13732817; doi:10.1002/jcsm.70415)
Supplement: Supplementary file 1 [file JCSM-17-e70415-s008.pdf]

Supporting Information Figures:

Remodeling of Skeletal Muscle during Iron Deficiency Anemia and Recovery after Intravenous Iron Therapy

Authors

Elke Pertler[1,2], Sonja A. Wagner[1,2], Marlene Panzer[2], Laura Obholzer[2], Ylena Sonnenburger[2], Maria R. Troppmair[2], Martina Saretto[2], Benedikt Schaefer[2], Claudia Manzl[3], Herbert Oberacher[4], Bettina Sarg[5], Klaus Faserl[5], Stefan Redl[6], Michael W. Hess[7], Theresia Telser[5], Jessica P. Popottnigg[2,8], Petra Gronich-Wondrak[2], Herbert Tilg[2], Heinz Zoller[1,2]

Affiliations

[1] Christian Doppler Laboratory for Iron and Phosphate Biology, Department of Internal Medicine I, Medical University of Innsbruck, Anichstrasse 35, 6020 Innsbruck, Austria

[2] Department of Internal Medicine I, Gastroenterology, Hepatology and Endocrinology, Medical University of Innsbruck, Anichstrasse 35, 6020 Innsbruck, Austria

[3] Institute of Neuropathology and Neuromolecular Pathology, Medical University of Innsbruck, Muellerstrasse 44, 6020 Innsbruck, Austria

[4] Institute of Legal Medicine and Core Facility Metabolomics, Medical University of Innsbruck, Muellerstrasse 44, 6020 Innsbruck, Austria

[5] Institute of Medical Biochemistry, Protein Core Facility, Biocenter, Medical University of Innsbruck, Austria

[6] Institute of Neuroanatomy, Medical University of Innsbruck, Innsbruck, Austria

[7] Institute of Histology and Embryology, Medical University of Innsbruck, 6020 Innsbruck, Austria

[8] Institute of Human Genetics, Medical University of Innsbruck, 6020 Innsbruck, Austria

Corresponding author: Heinz Zoller (e-mail: heinz.zoller@i-med.ac.at, telephone: +43 512 504 23397, fax: +43 512 504 27019)

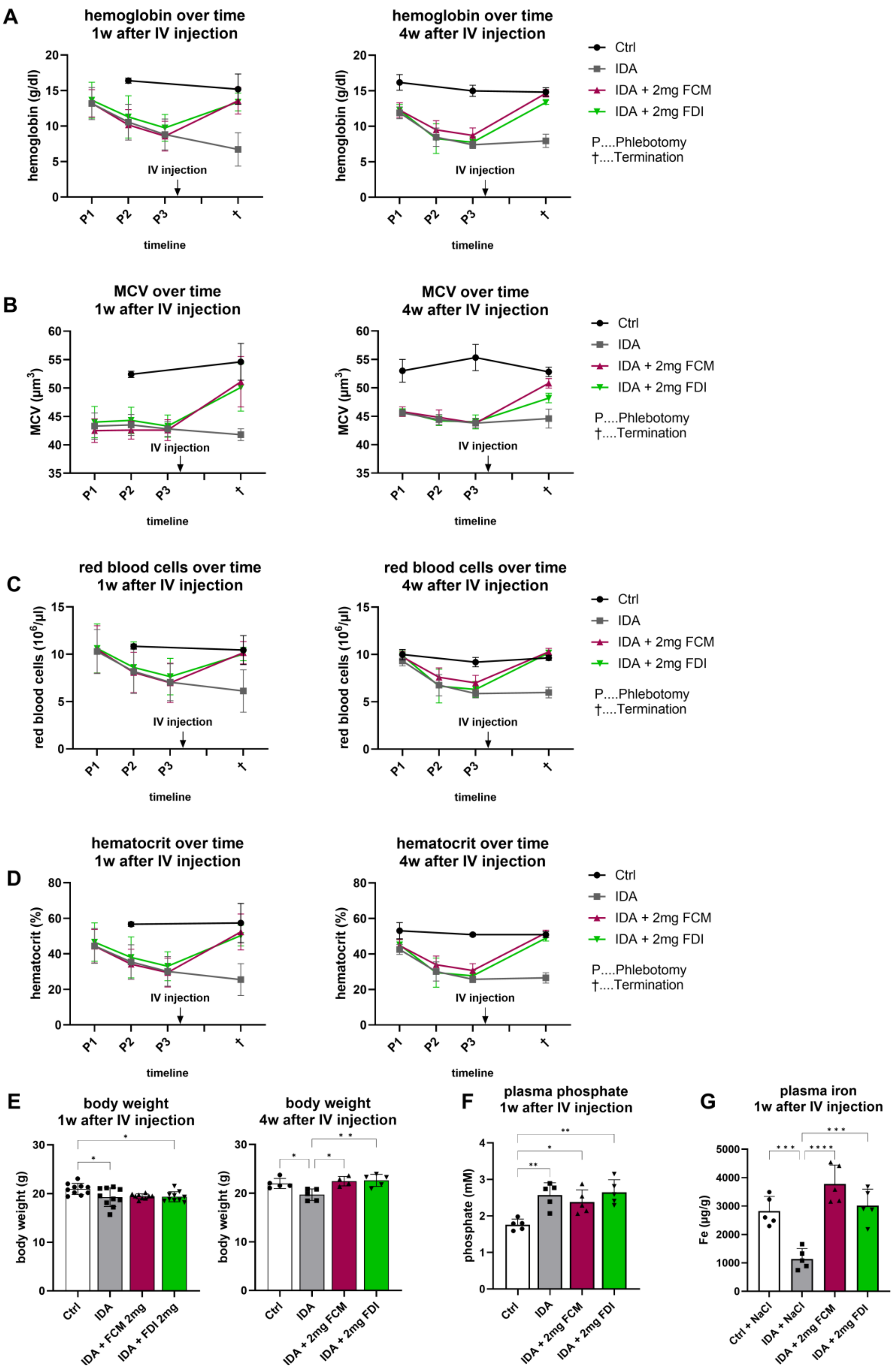

Figure S1 Characterization of the murine model of iron deficiency anemia

(A) Hemoglobin concentrations, **(B)** mean corpuscular volume (MCV), **(C)** red blood cells and **(D)** hematocrit are shown longitudinally during repeated phlebotomies (P) up to the termination point (†) of healthy controls (Ctrl), the 0.9% NaCl treated iron deficiency anemia (IDA) group and IDA mice treated with either FCM (IDA + 2 mg FCM) or FDI (IDA + 2 mg FDI) at one week (n=10) and at four weeks (n=5) following intravenous (IV) administration. Data are shown as mean ± SD. **(E)** Bar graphs show the body weight of all mice in Ctrl, IDA mice and IDA mice treated with FCM or FDI followed by measurements at one (n=10) and four weeks (n=5) after IV iron treatment. Data are presented as mean ± SD. Statistical differences among groups were assessed using one-way ANOVA, followed by Tukey's post hoc test. Plasma **(F)** phosphate and **(G)** iron concentrations measured by atomic absorption spectrometry of mice are shown one week after the IV administration (n=5). Statistical differences among groups were assessed using one-way ANOVA, followed by Tukey's post hoc test.
Parts of the data presented in this figure have been previously published [1].

Significance levels are denoted as: *p ≤ 0.05, **p < 0.01, ***p ≤ 0.001, ****p ≤ 0.0001.

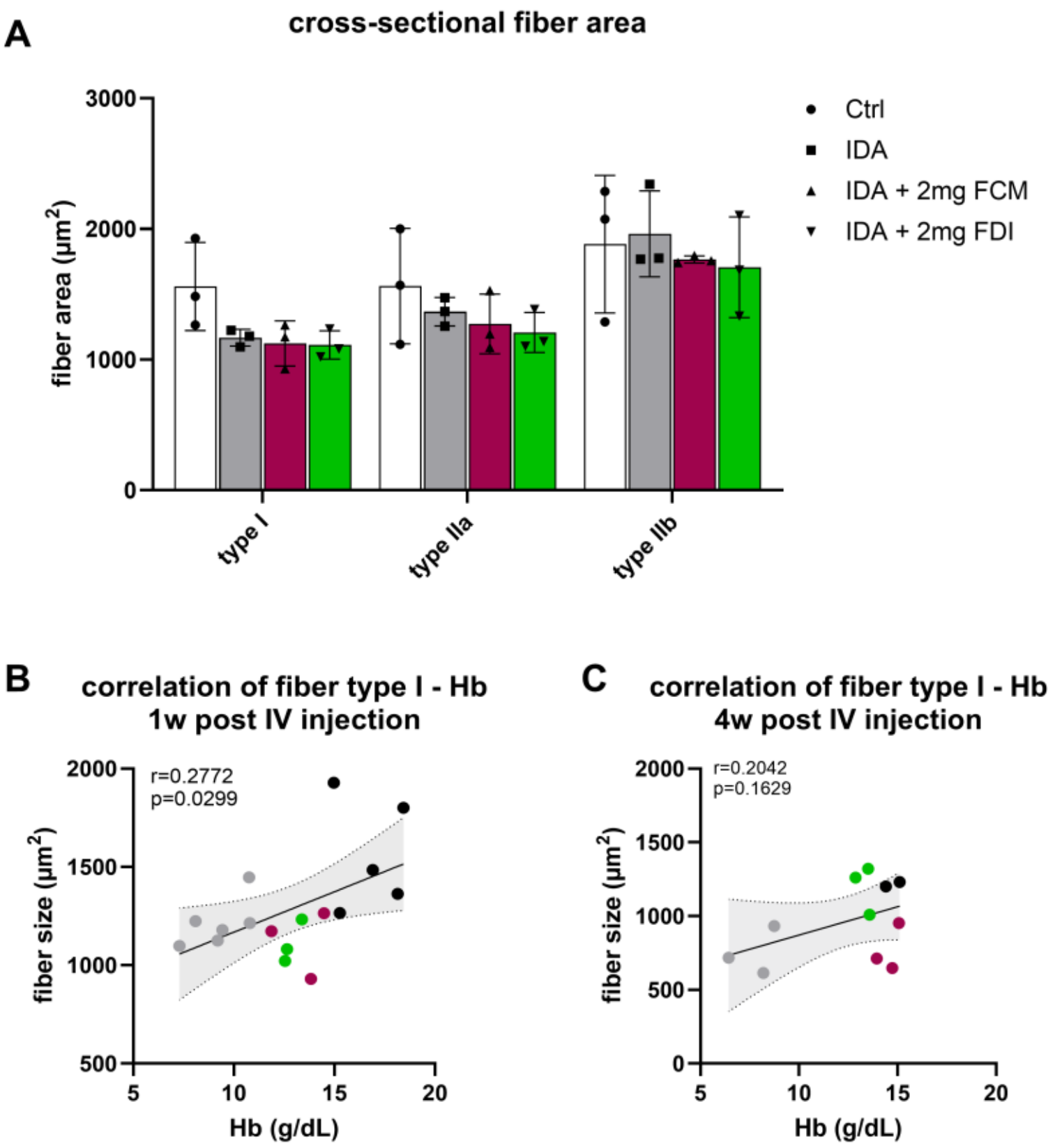

Figure S2 Cross-sectional diaphragm fiber area and its correlation with hemoglobin concentration in mice

(A) Cross-sectional area of diaphragm fiber types I, IIa and IIb in untreated controls (Ctrl), iron deficiency anemia (IDA) mice (n=3) and IDA mice treated for one week with intravenous (IV) ferric carboxymaltose (IDA + 2 mg FCM) or ferric derisomaltose (IDA + 2 mg FDI) (n=3 per

group). Data are shown as mean ± SD. Statistical differences were assessed by one-way ANOVA. **(B)** Correlation between type I fiber cross-sectional area and hemoglobin concentration one week after IV treatment in Ctrl (black, n=5), IDA (grey, n=6), FCM-treated (magenta, n=3) and FDI-treated mice (green, n=3). A simple linear regression with 95% confidence interval illustrates the overall relationship between fiber area and hemoglobin concentration. **(C)** Correlation between type I fiber cross-sectional area and hemoglobin concentration four weeks after IV treatment in Ctrl (black, n=2), IDA (grey, n=3), FCM-treated (magenta, n=3) and FDI-treated mice (green, n=3). A simple linear regression with 95% confidence interval illustrates the overall relationship between fiber area and hemoglobin concentration.

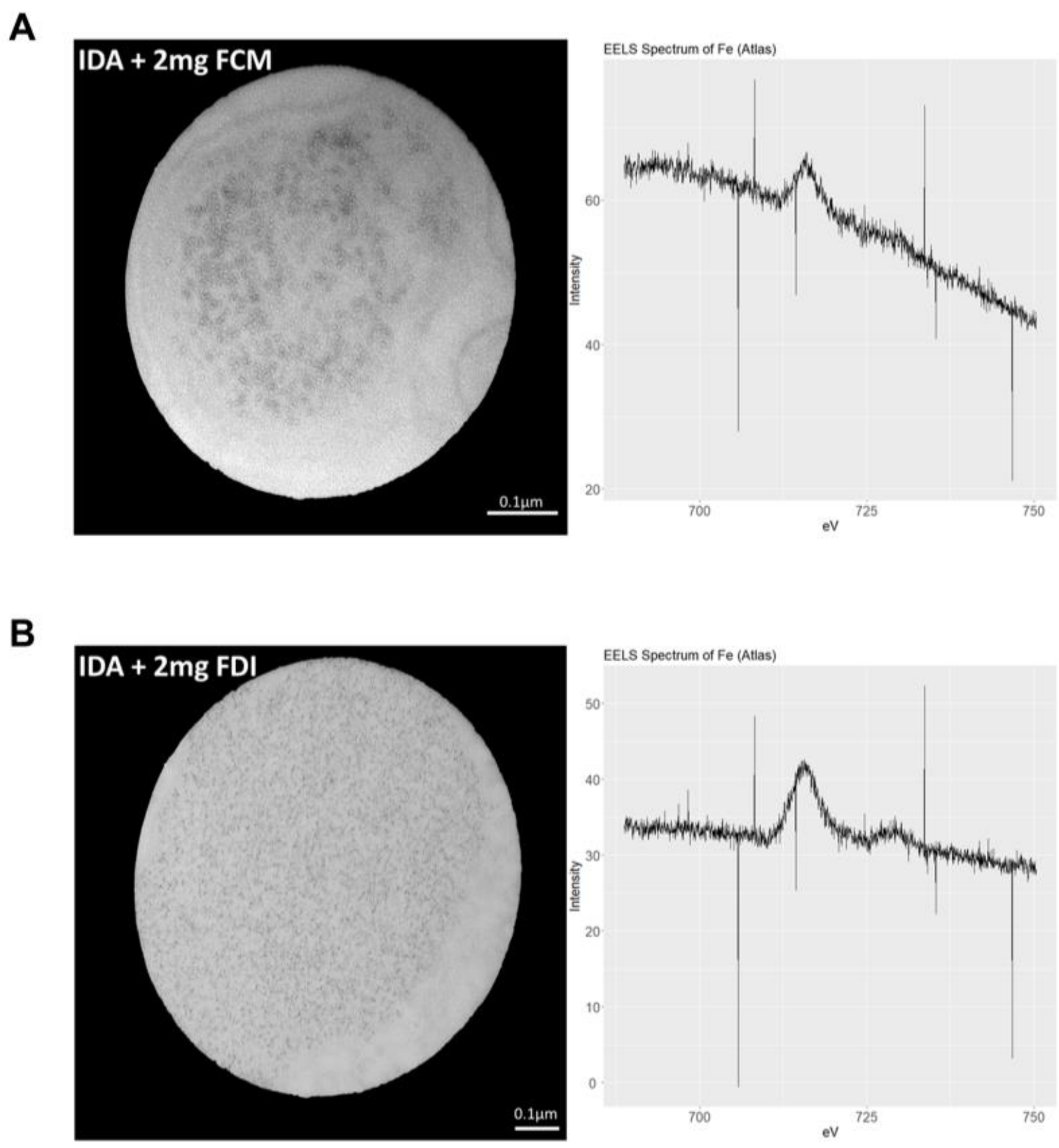

Figure S3 Electron energy loss spectroscopy (EELS) in skeletal muscle of iron treated mice

Electron microscopy and electron energy loss spectroscopy (EELS) analysis of electron-dense lysosomes in macrophages of skeletal muscle gastrocnemius in iron deficient anemic (IDA) mice one week after treatment with 2 mg of either **(A)** ferric carboxymaltose (FCM) or **(B)** ferric derisomaltose (FDI). EELS dhows iron (Fe). Scale bar represents 0.1 µm.

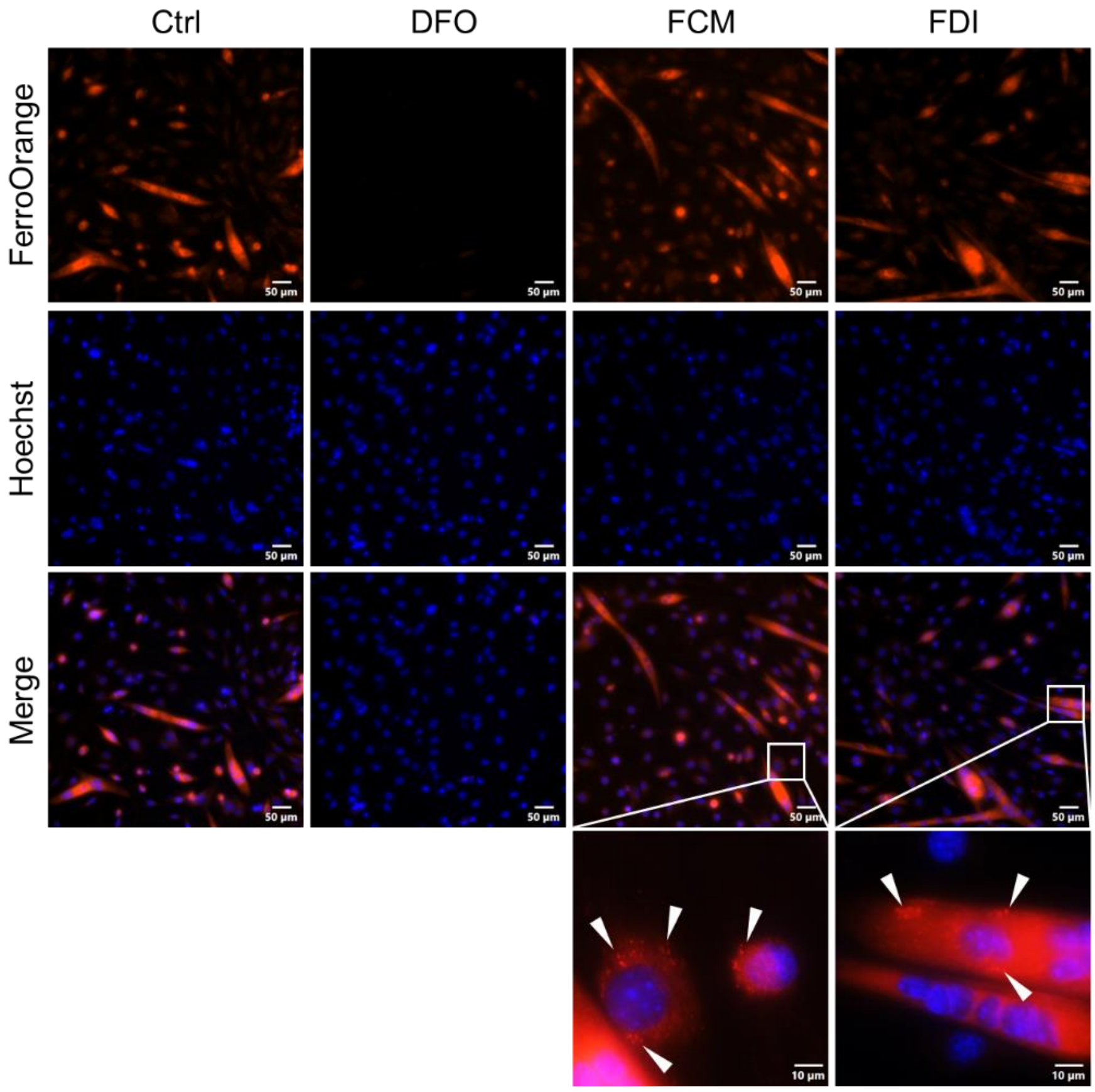

Figure S4 FerroOrange fluorescence staining of C2C12 myotubes

Representative images of FerroOrange staining in C2C12 myotubes differentiated for 9 days and pre-treated with 100 µM desferrioxamine (DFO) for 48 hours before cells were either left untreated or incubated in the presence of 200 µg/ml ferric carboxymaltose (FCM) or ferric derisomaltose (FDI) for 24 h (n=3 per group). Representative images of FerroOrange staining in FCM and FDI exposed C2C12 myotubes. Labile iron is represented in orange and shows a punctate intracellular staining of FCM and FDI-treated C2C12 cells in addition to the diffuse intracellular staining. Nuclei are stained with Hoechst shown in blue. Arrow heads indicate iron vesicles. Scale bar represents 50 µm and in the zoomed images 10 µm.

References

1. Wagner, S.A., et al., *Ferric carboxymaltose increases fracture risk in patients and reduces bone formation in mice with iron deficiency anemia.* Blood, 2026. **148**(1): p. 15-30.
