# Supplementary material for: Remodelling of Skeletal Muscle During Iron Deficiency Anaemia and Recovery After Intravenous Iron Therapy
Source: J Cachexia Sarcopenia Muscle. 2026 Nov 4;17(6):e70415. doi: 10.1002/jcsm.70415 (PMC13732817; doi:10.1002/jcsm.70415)
Supplement: Supplementary file 2 [file JCSM-17-e70415-s007.docx]

## **Supporting Information Methods: Detailed descriptions of experimental procedures and analytical methods used in this study.**

## **Remodeling of Skeletal Muscle during Iron Deficiency Anemia and Recovery after Intravenous Iron Therapy**

## **Animal experiments**

Directly after weaning mice were randomly assigned to the experimental groups. Iron deficiency anemia (IDA) animals were provided with a diet containing less than 10 mg Fe/kg (Ssniff GmbH, E15510-24), while control animals received a diet containing 168 mg Fe/kg (Ssniff GmbH, E15510-04) until the end of the experiment (Table S1) [1]. To reduce biological variability, experiments were conducted in female mice, reflecting the high prevalence of IDA among females of reproductive age. At the minimum age of six weeks, controlled phlebotomy was performed three times within one week before IV treatment. At the first phlebotomy, 300 µl of blood were collected and an equivalent volume of sterile 0.9% NaCl was immediately administered intraperitoneally. Hemoglobin concentrations were assessed after each phlebotomy and the volume of subsequent blood withdrawals was adjusted according to the hemoglobin concentration to ensure controlled induction of IDA while minimizing excessive blood loss. IV iron treatment with 2 mg of either ferric carboxymaltose (FCM; Ferinject by Vifor) or ferric derisomaltose (FDI; Monofer by Pharmacosmos) in a total volume of 200 µl diluted in 0.9% NaCl or with an equivalent volume of 0.9% NaCl in IDA and control animals. Mice received a single IV dose of 2 mg iron per mouse, corresponding to approximately 100 mg/kg body weight based on an average body weight of 20 g. This dose was selected based on previously established experimental protocols in mice and was intended to ensure robust systemic iron repletion after IDA induction and to allow comparison of tissue recovery and iron distribution after treatment with FCM or FDI. Due to species-specific differences in iron metabolism, pharmacokinetics and body-size scaling, this experimental dose was not intended to represent a direct human-equivalent dose. IV treatments were administered via the tail vein. Animals were monitored daily and pre-specified humane endpoints/exclusion criteria included impaired body condition, behavioral abnormalities, post-interventional hypothermia, failure to develop IDA and unsuccessful IV injection. All animals completing the experimental protocol with sufficient sample quality were included in the respective analyses. Collected blood samples were analyzed using the Vet abc^TM^ Animal Blood Counter, afterwards the plasma was collected and frozen at -80 °C until further processed.

Differences in muscular function were evaluated via the wire hang test conducted four weeks following intravenous iron administration. Mice were placed on a wire grid (43 × 43 cm; mesh size: 12 × 12 mm) and the latency to fall was recorded. Each animal underwent three trials, with rest intervals of at least 30 minutes between trials to prevent fatigue. Maximum hanging time and the mean duration were documented for subsequent analysis. A maximum trial duration of five minutes was imposed to minimize undue stress. Wire hang performance was normalized to body weight by calculating the holding impulse as latency to fall multiplied by body weight (g·s).

Tissues were harvested seven days or four weeks following IV treatment. An overview of the experimental procedures performed in each individual animal is provided in Supplementary Information Table S2. Parts of GAS, SOL, PLA and diaphragm (DIA) were snap frozen in liquid nitrogen or embedded in O.C.T. (Optimal cutting temperature) Compound Cryostat Embedding Medium (Scigen, 4586), frozen in isopentane cooled in liquid nitrogen and stored at -80 °C until further processed. For histological staining the skeletal muscles GAS, SOL and PLA were stored 4% formaldehyde solution (Liquid Productions GmbH) for paraffin slices.

## **Cell culture experiments**

C2C12 murine myoblasts were obtained from the Group of Physiology of Calcium Channels/Neuro-Muscular Physiology (Department of Physiology & Medical Physics at the Medical University of Innsbruck) and cultured according to the following standard protocols. Cells were maintained in Growth Medium (GM) consisting of Dulbecco’s Modified Eagle Medium (DMEM) – GlutaMAX (Gibco™, 10566016), supplemented with 10% fetal bovine serum (FBS) (Gibco™, A5256701) and 1% Penicillin and Streptomycin (P/S) (Gibco™, P4333) for proliferation. Cells were incubated at 37 °C with 5% CO_2_. After reaching 60% confluency, cells were detached using Trypsin (Gibco™, 25300054) and passaged up to passage 30.

For differentiation, 3 × 10^4^ cells were seeded into 6-well plates. At 90% confluency, the medium was replaced with differentiation medium (DM), which contained DMEM – GlutaMAX supplemented with 1% horse serum (HS) (Gibco™, 16050122) and 1% P/S. Differentiation proceeded for 9 days, with DM replaced every other day. To induce iron deficiency, 100 µM desferrioxamine (DFO) was added on day 7 of differentiation for 24 hours. Media was removed and cells were washed two times with PBS to remove DFO. Subsequently, cells were treated for another 24 hours with either 100 µM DFO or with 200 µg/ml FCM or FDI in serum-free DM. Cells were collected according to the following methods as described below.

## **Mitochondrial stress test**

Oxygen consumption rate (OCR) was assessed using the Seahorse XF HS Mini Analyzer (Agilent Technologies). 3 × 10^3^ cells per well were seeded in Agilent Seahorse XF HS Mini cell culture microplates, differentiated and treated with DFO or with IV iron formulations as described previously. On day 9, cells were incubated for 45 minutes at 37 °C in a CO_2_-free incubator with Seahorse XF DMEM assay medium (pH 7.4) (Agilent, 103575-100), supplemented with 10 mM glucose (Agilent, 103577-100), 1 mM pyruvate (Agilent, 103578-100) and 2 mM glutamine (Agilent, 103579-100).

OCR was measured using the Seahorse XF Cell Mito Stress Test Kit (Agilent, 103010-100) following the manufacturer’s protocol. After three baseline measurements, 1.5 µM oligomycin was injected to inhibit ATP-linked respiration. Subsequently, 2 µM FCCP (carbonyl cyanide-4-(trifluoromethoxy) phenylhydrazone) was added to uncouple mitochondrial respiration and determine the maximal respiratory capacity. Finally, 0.5 µM rotenone and antimycin A was applied to inhibit complex I and assess non-mitochondrial respiration. OCR values were normalized to cell number, determined by nuclear staining with Hoechst and automated image-based quantification of nuclei.

## **RNA isolation, real-time qPCR**

For tissue samples, small pieces were immersed in 1 ml TRI Reagent^®^ (Sigma-Aldrich^®^, T3934) and mechanically homogenized using the Precellys^®^ homogenization system (VWR, 432-0351, 432-0355). Cell samples were directly lysed in 1 ml TRI Reagent^®^ without additional homogenization. The aqueous phase containing RNA was transferred to a new tube, mixed with 400 µl ethanol and loaded onto Monarch^®^ RNA Cleanup columns (New England Biolabs, T2030L). RNA purification was performed according to the manufacturer’s instructions and RNA was eluted in 10 µl nuclease-free water. 2 µg of total RNA was reverse-transcribed into complementary DNA (cDNA) using the High-Capacity cDNA Reverse Transcription Kit (Applied Biosystems™, 4368813) according to the manufacturer’s protocol. For G/C-rich transcripts, RNA was preheated with 10× RT Random Primers at 95 °C for 5 min before the addition of the reverse transcription mix.

Quantitative real-time PCR (RT-qPCR) was performed using SYBR™ Select Master Mix (Applied Biosystems™, 13246519) with primers listed in table 1. Reactions were carried out according to the manufacturer’s instructions. Relative mitochondrial gene expression levels were calculated using the ΔΔCT method, normalizing to Rpp30 and to control group values.

Table 1: Murine primer sequences for RT-qPCR analysis

| **Gene** | **Forward primer** | **Reverse primer** |
| --- | --- | --- |
| *Tfrc* | 5′–TTGGATTCATGAGTGGCTAC–3′ | 5′–AGTGTTTTGAGGTCTGCCC–3′ |
| *Ldha* | 5′–CAACCCTCAAGGACCAGCTG–3′ | 5′–CATCCGCCAAGTCCTTCATT–3′ |
| *Zip14* | 5′–CTGGCTATTGGTGCCTCCTTCA–3′ | 5′–TGCCAGCATTGAGCAGGATGAC–3′ |
| *Dmt1* | 5′–TTGCAGCGAGACTTGGAGTGGT–3′ | 5′–GCTGAGCCAATGACTTCCTGCA–3′ |
| *Tbp* | 5′–GGAGCCAAGAGTGAAGAAC–3′ | 5′–GGGAACTTCACATCACAGC–3′ |
| *CoxI* | 5′–GCTCAATCTGCTTACGCCA–3′ | 5′–CTGTGGATCCGTTCGTAGT–3 |
| *Rpp30* | 5′–GCGGACTCTGGGTTGAAGTC–3′ | 5′–GCTCTGCAATTTGTGGACACG–3 |

Detection and data acquisition were conducted on a StepOnePlus™ Real-Time PCR System (Applied Biosystems™, 272002967). Genomic DNA (gDNA) of cells was isolated with the Spin gDNA Extraction Kit according to the instructions of the manufacturer (Monarch^®^, #T3010). RT- qPCR was performed with *Rpp30* and *CoxI*.

## **Western blot**

Tissue samples were mechanically homogenized in 400 µl RIPA Lysis and Extraction Buffer (Thermo Scientific™) supplemented with 1× Halt™ Protease Inhibitor Cocktail (Thermo Scientific™) and 1× Halt™ Phosphatase Inhibitor Cocktail (Thermo Scientific™) using the Precellys^®^ homogenization system (VWR, 432-0351, 432-0355). Cells were harvested in 150 µl RIPA buffer with the inhibitors and lysed by sonication. Proteins were quantified using the Pierce™ Dilution-Free™ Rapid Gold BCA Protein Assay Kit (Thermo Scientific™), following the manufacturer’s instructions.

Samples were mixed with 6× Laemmli SDS sample buffer (Thermo Scientific™, #J61337.AD) and incubated for 5 min at 50 °C. Equal amounts of protein were resolved by SDS–PAGE using 10% Mini-PROTEAN^®^ TGX Stain-Free™ gels (BioRad, 4568036) at 100 V for 1 h and transferred onto PVDF membranes (Immobilon, Merck Millipore) at 100 V at 37 °C. Membranes were blocked for 1 h in blocking buffer (3.3% milk powder, 0.1% Tween-20, 4% 25× PBS) and incubated overnight at 4 °C with primary antibodies: OxPhos Rodent WB Antibody Cocktail (Invitrogen™, 45-8099; 1:250) and Vinculin (Cell Signaling, 4650; 1:10,000). Following three washes with wash buffer (0.3% milk powder, 0.1% Tween-20, 4% 25× PBS), membranes were incubated for 1 h with HRP-conjugated secondary antibodies: Goat anti-Rabbit IgG-HRP (Agilent Dako, P0448; 1:2000) or Rabbit anti-Mouse IgG-HRP (Agilent Dako, P0260; 1:1000). Antibody specificity was assessed by detection of bands at the expected molecular weight and by the absence of major unspecific bands. All antibodies were used according to validated applications provided by the manufacturer and/or previous literature. Detection was performed using the SuperSignal™ West Dura Extended Duration Substrate (Thermo Scientific™) and signal was visualized with a ChemiDoc™ XRS+ imaging system (Bio-Rad).

## **L-Lactate assay**

L-lactate concentrations were determined in cell culture medium. Medium was filtered using a 10 kDa Amicon^®^ centrifugal filter at 4,000 × g for 15 minutes at 4°C. L-lactate levels were measured using the L-Lactate Assay Kit (Abcam, #ab65330) according to the manufacturer’s instructions for colorimetric detection with a microplate reader (TECAN Spark, Tecan Trading AG).

## **Proteomic analysis**

Gastrocnemii were homogenized in 500 µl ammonium bicarbonate buffer (ABC buffer; 100 mM, pH 8.0) supplemented with 0.5% sodium deoxycholate. Homogenization was performed using a Precellys Evolution system equipped with 2.8 mm metal beads for three 30 s cycles. Protein concentrations were determined by Bradford assay (ROTI®-Nanoquant K880, Carl Roth). Proteins were reduced by adding 50 µl of 100 mM dithiothreitol (DTT) in ABC buffer and incubating at 56 °C for 30 min. Samples were subsequently digested with sequencing-grade modified trypsin (Promega, P/N V5111) at an enzyme-to-substrate ratio of 1:85 for 16 h at 37 °C. Alkylation of free cysteines was achieved by adding 50 µl of 550 mM iodoacetamide in ABC buffer and incubating for 20 min at room temperature in the dark with gentle agitation. Aliquots corresponding to 100 µg peptide material were buffer-exchanged into 100 µl of 100 mM triethylammonium bicarbonate (TEAB, pH 8.5) using Pierce Peptide Desalting Spin Columns (Thermo Scientific, P/N 89852). Samples were labeled with TMTpro™ 16plex reagents (Thermo Scientific, P/N A44521) following the manufacturer’s protocol. All labeled samples were pooled, lyophilized to dryness and reconstituted in 90 µl of 0.1% formic acid. Peptides were fractionated under high-pH reversed-phase conditions using an XBridge Peptide BEH C18 column (4.6 mm × 250 mm, 300 Å, 5 µm; Waters, P/N 186003625), as described previously [2]. The resulting fractions were combined into 11 pools, lyophilized and stored at −20 °C. For LC–MS analysis, peptides were reconstituted in 0.1% formic acid and 2% of each pool was injected.

Peptide digests were analyzed on an UltiMate 3000 nano-HPLC system coupled to an Orbitrap Eclipse mass spectrometer (Thermo Scientific) equipped with a Nanospray Flex ion source, as previously described [2]. Raw data were processed using Proteome Discoverer v2.5 (Thermo Scientific). MS/MS spectra were searched with the Sequest HT engine against the UniProt mouse reference proteome (last modified 27 April 2021). The false discovery rate (FDR) for peptide and protein identification was restricted to 1%. Quantitative protein fold changes were derived from TMTpro reporter ion intensities extracted in MS^2^ scans using default software settings (Supporting information Table S3).

*Statistical analysis*

Differential protein abundance between groups was assessed using a two-sided Welch's t-test. Volcano plots display log_2_ fold changes against the corresponding −log_10_ nominal p-values (Supporting information Table S4 – S5). Proteins were filtered to include only high-confidence protein identifications detected in both proteomic acquisition runs. Proteins with an absolute log_2_ fold change > 0.5 were considered to exhibit biologically meaningful regulation. Benjamini-Hochberg correction for multiple testing was performed but did not identify statistically significant proteins after adjustment, likely reflecting the relatively small sample size in relation to the number of quantified proteins. Therefore, volcano plots are based on nominal p-values combined with biologically relevant effect sizes. Gene Ontology (GO) enrichment of Biological Processes was performed using STRING (version 12.0; https://string-db.org). Proteins showing biologically meaningful regulation (log_2_ fold change < −0.5 for IDA vs. Ctrl or > 0.5 for FDI vs. FCM) were used as input for the respective enrichment analyses. Functional enrichment was calculated using Fisher’s exact test with Benjamini–Hochberg correction for multiple testing, and GO terms with an FDR < 0.05 were considered as significantly enriched (Supporting information Table S6 – S7). Protein-protein interaction network of proteins upregulated after FDI were generated in STRING using the same protein set employed for GO enrichment analysis. A minimum required interaction score of 0.4 (medium confidence) was applied. Network clustering was performed using k-means clustering (k=3). Network nodes correspond to proteins, where splice isoforms and post-translational modifications are consolidated, such that each node represents all protein products encoded by a single gene locus. GO enrichment results were visualized in R (version 2026.01.1+403, R Foundation for Statistical Computing, Vienna, Austria) as dot plots, displaying the eight most strongly enriched GO Biological Process terms ranked by enrichment strength.

## **Quantitative analysis of sugars, phosphosugars and short-chain carboxylic acids in homogenized tissue samples**

The quantitative analysis was performed following a published protocol [3].

*Chemicals*

Methanol, acetonitrile and water (all HPLC grade) were obtained from Honeywell (Seelze, Germany). 1-ethyl-3-(3-dimethylaminopropyl)carbodiimide (EDC), 3-nitrophenylhydrazine (3-NPH) and pyridine as well as standards of glucose, glucose 6-phosphate, dihydroxyacetone phosphate, phosphoenolpyruvic acid, pyruvic acid, lactic acid, ribose 5-phosphate, citric acid, α-ketoglutaric acid, succinic acid, fumaric acid, malic acid and oxaloacetic acid were purchased from Sigma-Aldrich (St. Louis, MO, USA). The stable isotope labeled standards were provided by Sigma-Aldrich (glucose 6-phosphate-^13^C_6_) or Toronto Research Chemicals (Toronto, Ontario, Canada). Stock solutions of standards (10 mg/ml) and internal standards (1 mg/ml) were prepared in acetonitrile-water (1:1, v/v). For the preparation of calibration standards, the reference standard solutions were mixed and dissolved in acetonitrile-water (1:1, v/v) to reach concentrations between 10 ng/ml and 100 µg/ml.

*Derivatization procedure*

10 µl of the processed tissue samples or calibration standards were pipetted into a 1.5 ml vial and mixed with 5 µl of the internal standard solution (10 µg/ml of each internal standard in acetonitrile-water (1:1, v/v)) as well as 50 µl of an EDC solution (10 mg/ml in acetonitrile-water (3:1, v/v)), 50 µl of a 3-NPH solution (10 mg/ml in acetonitrile-water (3:1, v/v)) and 5 µl of pyridine. Derivatization was performed at room temperature for 30 min, after which, the excess reagent was removed by drying at 60 °C with a gentle stream of nitrogen. The dried samples were dissolved with 300 µl of water-methanol (3:1, v/v).

*Liquid chromatography-tandem mass spectrometry*

The LC-MS/MS system consisted of an Acquity Premier UPLC System (Waters, Milford, MA, USA) and a QTrap 6500+ mass spectrometer (Sciex, Framingham, MA, USA). Separations were accomplished on a Kinetex biphenyl column (2.6 µm, 100 Å, 100 × 2.1 mm; Phenomenex, Aschaffenburg, Germany) using a ten-minute gradient of 2-85% acetonitrile in aqueous 0.5% acetic acid solution. The flow rate was set to 200 µl/min and the column temperature was kept at 50 °C. The injection volume was 10 µl. Mass spectrometry detection was performed with electrospray ionization in positive ion mode. Multiple reaction monitoring was carried out using the precursor-to-product ion transitions summarized in Table 2. The peak area ratios of analyte-specific ions to the corresponding internal standard ions obtained from the calibration standards versus concentrations were used to fit 1/x−weighted linear, least squares regression models. The calibration models were used to calculate the analyte concentrations from the peak area ratios of analyte-specific ions to the corresponding internal standard ions obtained from the samples (Supporting information Table S8).

Table 2 Multireaction monitoring transitions for the targets and the corresponding internal standards

| **Target** | **Q1 (*m/z*)** | **Q3 (*m/z*)** | **Internal Standard** |
| --- | --- | --- | --- |
| Glucose | 314 | 137 | Glucose-^13^C_4_ |
| Glucose 6-phosphate | 529 | 232 | Glucose 6-phosphate-^13^C_6_ |
| Dihydroxyacetone phosphate | 439 | 232 | Dihydroxyacetone phosphate-^13^C_2_ |
| Phosphoenolpyruvic acid | 437 | 214 | Phosphoenolpyruvic acid-^13^C_3_ |
| Pyruvic acid | 357 | 92 | Pyruvic acid-^13^C_1_ |
| Lactic acid | 224 | 122 | Lactic acid-^13^C_3_ |
| Ribose 5-phosphate | 499 | 232 | Phosphoenolpyruvic acid-^13^C_3_ |
| Citric acid | 596 | 401 | Citric acid-^13^C_6_ |
| α-Ketoglutaric acid | 550 | 371 | α-Ketoglutaric acid-D_6_ |
| Succinic acid | 387 | 234 | Fumaric acid-^13^C_4_ |
| Fumaric acid | 385 | 232 | Fumaric acid-^13^C_4_ |
| Malic acid | 403 | 208 | Malic acid-D_3_ |
| Oxaloacetic acid | 536 | 357 | α-Ketoglutaric acid-D_6_ |
| Glucose-^13^C_4_ | 320 | 137 |  |
| Glucose 6-phosphate-^13^C_6_ | 534 | 232 |  |
| Dihydroxyacetone phosphate-^13^C_2_ | 442 | 232 |  |
| Phosphoenolpyruvic acid-^13^C_3_ | 440 | 232 |  |
| Pyruvic acid-^13^C_1_ | 358 | 137 |  |
| Lactic acid-^13^C_3_ | 227 | 137 |  |
| Citric acid-^13^C_6_ | 602 | 137 |  |
| α-Ketoglutaric acid-D_6_ | 556 | 137 |  |
| Fumaric acid-^13^C_4_ | 389 | 137 |  |
| Malic acid-D_3_ | 406 | 137 |  |

## **Non-target analysis of lysed cell culture samples**

*Chemicals and reagents*

Water, methanol (MeOH) and formic acid (FA) were obtained from Honeywell (Seelze, Germany). Internal standards (fencamfamine and heptobarbital) and reference standards used to prepare quality control samples were provided by Lipomed (Arlesheim, Switzerland), Cerilliant (Round Rock, TX, USA), or Sigma-Aldrich (Saint Louis, MO, USA). An overview on the compounds is provided in Supporting information Table S9.

*Sample preparation*

150 µl of lysed cell culture sample were mixed with internal standards (fencamfamine, 10 µl, 60 ng/ml; heptobarbital, 2 µl, 100 ng/ml). The mixture was evaporated to dryness at 25 °C under a gentle stream of nitrogen. The dry residue was reconstituted in 50 µl of water-methanol (3:1, v/v) for LC-MS/MS analysis.

*Non-targeted LC-MS/MS analysis*

The LC-MS/MS system consisted of a Vanquish Flex Duo UHPLC System (Thermo Scientific, Waltham, MA, USA) coupled to an Orbitrap Exploris 240 mass spectrometer (Thermo Scientific). The chromatographic separation was carried out on a Kinetex biphenyl column (2.6 μm, 100 Å, 100 × 2.1 mm; Phenomenex) using a 15-minute linear gradient of 5-95% methanol in aqueous 0.1% formic acid solution (v/v). The temperature of the column was constant at 50 °C and the applied flow rate was 250 µl/min. 10 µl of the sample were injected. The mass spectrometer was operated either in positive or negative electrospray ionisation (ESI) mode with the H-ESI source. The spray voltage was set to 3.5 kV or -2.5 kV, respectively. Gas flows of 50 arbitrary units for the sheath gas, 10 arbitrary units for the aux gas and 1 arbitrary unit for the sweep gas were employed. The temperature of the ion transfer tube was adjusted to 325 °C. The vaporizer temperature was 350 °C. The instrument was operated at a mass resolution of 60,000 for MS and 30,000 for MS/MS and automatically recalibrated before sample injection with the internal mass calibration system. The scan range was *m/z* 105-1000 for MS and started at *m/z* 50 for MS/MS. A duty cycle in the data-dependent acquisition mode included a single MS scan followed by eight dependent MS/MS scans (maximum injection time, 100 ms each). MS/MS spectra were acquired at 15, 25 and 35 eV. Former target ions were excluded for 30 seconds after two occurrences.

*Quality control*

Quality control included the analysis of different kinds of control samples. These included a system suitability test sample (aqueous solution of reference compounds, 10 ng/ml each), a pooled study sample and blanks (procedural blanks and solvent blanks). Monitoring retention times, peak areas and *m/z*-values of the reference compounds and the internal standard across samples provided information on the stability and reproducibility of the LC-MS/MS system-performance. The individual controls were analysed before starting the sample batch and in between each set of twelve samples.

*Compound annotation*

Positive ion mode and negative ion mode runs were analyzed separately. MSforID Search and Compound Discoverer 3.3 SP2 (Thermo Scientific) were employed for compound annotation.

Compound annotation with MSforID Search involved conversion of tandem mass spectral data in an open format, automated library search and expert reviewing [4]. Raw data files in .raw format were converted into the “mascot generic format” (mgf) using the rawrr package [5] available in R (R Foundation for Statistical Computing, Vienna, Austria). Library search was accomplished with MSforID Search (17) written in R (R Foundation for Statistical Computing) employing packages developed by the RforMassSpectrometry initiative [6]. The acquired tandem mass spectra were matched to reference spectra using the following settings: ‘*m/z* tolerance’ ± 0.01 and ‘intensity threshold value’ 0.01. Employed tandem mass spectral libraries included the Wiley Registry of Tandem Mass Spectral Data, MassBank and NIST 11 MS/MS Library. A library search match was considered as putatively correct if the precursor ion mass error was within 10 ppm of the predicted ion mass and the average match probability value was greater than 4.0. Finally, the correctness of tentative identifications was checked by expert reviewing. For this purpose, the corresponding tandem mass spectra were written to a single file and imported into the MS Search program (version 2.0 g, National Institute of Standards and Technology, Gaithersburg, MA, USA).

In Compound Discoverer, the Input File node was used to submit the files. The Select Spectra node was used with open settings and a default 1.5 S/N threshold. The ChromAlign node was used to align chromatographic peaks in all files. The Detect Compounds node was used with 5 ppm mass tolerance, 10,000 minimum peak intensity, at least 5 scans per peak, peak detection S/N threshold 1.5, remove baseline false, gap ratio threshold 0.35, max peak width 1 min and compound detection of [M+H]^+^ ions as base ions for positive mode as well as [M-H]^−^ ions for negative mode. The Group Compounds node was used with 5 ppm mass tolerance, 0.2 min retention time (RT) tolerance. The Fill Gaps node was used with 5 ppm mass tolerance. The Search ChemSpider node was used with 5 ppm mass tolerance. The databases selected included the Serum Metabolome Database, BioCyc and KEGG. The Search mzCloud node was used to compare measured MS/MS spectra with the HighChem HighRes algorithm at precursor mass and FT fragment mass tolerances of 10 ppm. The Apply mzLogic node was set with 10 ppm mass tolerance. Compound annotation was primarily accomplished based on the mzCloud Search results.

For statistical analysis, only putatively annotated compounds were considered. These were 115 features in positive mode (Supporting information Table S10) and 49 features in negative mode (Supporting information Table S11). The annotated compounds belonged to the following classes of compounds: amino acids, biogenic amines, nucleic acid components, vitamins, acylcarnitines, organic acids, sugar phosphates, fatty acids as well as lipids. The peak areas of the features in the extracted ion chromatograms were obtained with TraceFinder software (Thermo Scientific).

*Statistical evaluation*

Statistical evaluation was conducted using the MetaboAnalyst 6.0 platform for metabolomics data analysis [7]. (https://www.metaboanalyst.ca). Raw metabolite intensities were filtered by low variance using a 5% interquartile range cutoff and by low abundance based on mean intensity. Data were normalized using reference standards, log_10_-transformed and auto-scaled prior to multivariate analyses. Principal component analysis (PCA) and hierarchical clustering heatmap were generated to assess global metabolic variation and identify differentially abundant metabolites. For pairwise differential metabolite abundance analysis, normalized metabolite abundance data were exported and analyzed in Excel (Supporting information Table S12). Data were log_2_-transformed, and log_2_ fold changes of IDA to Ctrl were calculated as the difference between group means (Supporting information Table S13). Raw p values were calculated using two-sided unpaired t-tests, and -log_10_-transformed p-values were used for volcano plot visualization. Benjamini-Hochberg FDR-adjusted p-values were calculated for each pairwise comparison. Fold-change thresholds and significance cutoffs were applied to identify differentially abundant metabolites. Top 50 of significantly up- and down-regulated metabolites identified from volcano plot outputs were subsequently used for KEGG pathway enrichment within MetaboAnalyst (Supporting information Table S14).

## **RNA-seq analysis**

RNA samples were shipped to Novogene (UK) for library preparation and paired-end sequencing on an Illumina platform. Libraries were generated using poly(A) enrichment, followed by cDNA synthesis, fragmentation and adaptor ligation. Sequencing quality control, read alignment and downstream data processing were carried out by Novogene using their standardized bioinformatics pipeline.

## **Immunofluorescence staining**

Diaphragms embedded in OCT compound were cryosectioned at 8 µm thickness using a cryostat (LEICA Biosystems, CM1950) at −20 °C. Following the protocol of Dyar et al. [8], skeletal muscle was stained for different fiber types. Sections were air-dried at room temperature and washed three times with 1× PBS. Unless otherwise specified, all washing steps were performed at room temperature in 1× PBS. Non-specific binding was blocked with 10% goat serum for 1 h at room temperature, followed by incubation with Mouse on Mouse (M.O.M.) IgG Blocking Reagent (Invitrogen™, R37621) overnight at 4 °C. Sections were subsequently washed and incubated for 1 h at 37 °C with primary antibodies specific for myosin heavy chain isoforms, all obtained from the Developmental Studies Hybridoma Bank (DSHB): MyHC-I (clone BA-D5; 1:50), MyHC-IIa (clone SC-71; 1:100) and MyHC-IIb (clone BF-F3; 1:100). Following three washes, sections were incubated for 45 min at 37 °C in the dark with fluorophore-conjugated secondary antibodies: goat anti-mouse IgG1 Alexa Fluor 488 (Invitrogen™, A-21121; 1:2000), goat anti-mouse IgG2b Alexa Fluor 647 (Invitrogen™, A-21242; 1:2000) and goat anti-mouse IgM Alexa Fluor 549 (Invitrogen™, A-21044; 1:2000). Antibody specificity was assessed by including no-primary antibody controls to exclude unspecific secondary antibody binding and by evaluating the expected staining patterns. All antibodies were used according to validated applications provided by the manufacturer and/or previous literature. After final washes, slides were mounted using a DAPI-containing antifade mounting medium (Abcam, ab104139). Images were acquired using an Olympus VS120 slide scanner at 20× magnification. Fiber size quantification was performed by investigators blinded to group allocation.

## **Histological staining**

Paraffin embedded gastrocnemii were sliced in 5 µm sections. Sections were deparaffined and hydrated through graded ethanol. Sections were incubated for one hour at 37 °C with 1% HCl and 20% of either potassium ferrocyanide (II) (^©^MORPHISTO GmbH, 13306) for ferric iron (Perls’ staining) or potassium ferricyanide (III) (Roth^®^, 7971) for ferrous iron (Turnbull’s Blue stain). After washing five times with A. dest., slides were blocked for one hour in peroxidase blocking buffer (methanol containing 0.01M sodium azide and 0.03% H_2_O_2_) followed by washing three times in 0.1 M Sørensen phosphate buffer (pH 7.4). Enhancement of the staining was performed by incubating the sections in 0.025% 3,3'-Diaminobenzidine tetrahydrochloride (DAB) (Roth^®^, CN75) and 0.005% H_2_O_2_ (Roth^®^, 8070) in 0.1 M Sørensen phosphate buffer (pH 7.4). The reaction was stopped by washing briefly in tap water after staining. Nuclear staining was performed using Mayer’s hematoxylin (^©^MORPHISTO GmbH, 16133) following rinsing thoroughly in tap water to allow blueing. Slides were rehydrated in graded ethanol and cover slipped in xylene. Histological analyses and image quantification were performed by investigators blinded to group allocation.

## **Live Cell Imagining in C2C12 myotubes**

For visualization of intracellular ferrous iron, differentiated C2C12 myotubes were briefly washed with chilled PBS and incubated in serum-free DMEM containing FerroOrange (Dojindo, #F374-10; 1:1000). Nuclei were simultaneously labeled with Hoechst 33342 (Invitrogen™, #33342; 1:2000). Following a 45-min incubation, live-cell fluorescence microscopy was performed on an Olympus IX83 imaging platform (Olympus Corporation, Japan) using a 20× objective.

## **Electron microscopy**

Gastrocnemii of mice treated as illustrated in Figure 1F were dissected and fixed immediately with buffered glutaraldehyde, followed by aqueous 0.5% OsO_4_, dehydrated and embedded in Epon epoxy resin [1]. The same standard procedure was used for C2C12 myoblasts. Transmission (TEM) analysis was performed on a Philips CM120 at 80 kV and electron energy loss spectroscopy (EELS) was performed on a ZEISS Libra 120, operated at 80 kV. For EELS analysis sections were cut at 55 nm Leica Ultracut S ultramicrotome and picked up on 400 G copper grids. EELS was performed via the PEELS plugin of ImageSP software (Version 1.2.14.49) at magnifications of 25k×-55k×.

## **Atomic absorption spectrometry**

Tissue samples were digested in 750 µl of ultrapure nitric acid (67-70%, OptimaTM grade, Thermo Fisher Scientific) using a high-performance microwave (MARS 6, CEM Microwave Technology). Iron content was quantified by GF-AAS (M6 Zeeman GFAA-Spectrometer, Thermo Scientific) employing Extended Lifetime Graphite Cuvettes (Thermo Fisher Scientific). Measurements were performed at a wavelength of 248.3 nm with 0.2 nm bandpass, using Zeeman background correction. Ash and atomization temperatures were set to 1100 °C and 2100 °C, respectively, under an argon atmosphere. Calibration solutions (0.5-12.5 µg/L) were prepared by serial dilution of a 1000 mg/l iron standard (TraceCERT, Sigma-Aldrich) in 0.25% ultrapure nitric acid. Samples were normalized to the wet weight.

## **Phosphate measurement**

Plasma inorganic phosphate concentrations 1 week after IV iron treatment were measured colorimetrically as previously described [1]. Briefly, after protein precipitation with 20% trichloroacetic acid, phosphate was quantified based on the reduction of phosphomolybdate by ascorbic acid to molybdenum blue. Absorbance was measured at 820 nm after incubation for 20 min at 45 °C, and phosphate concentrations were calculated from potassium dihydrogen phosphate standards.

## **Muscle strength measurements in patient cohort**

For this study two cohorts were combined (ClinicalTrials.gov identifiers: NCT03238911 and NCT03237065) and reanalyzed [9]. Maximal inspiratory pressure (MIP) and maximal expiratory pressure (MEP) analyses were assessed as non-invasive measures of respiratory muscle strength, following the ATS/ERS guidelines from 2002. MIP reflects diaphragm and other inspiratory muscle strength, while MEP reflects abdominal and other expiratory muscle strength. Measurements were performed using a MicroRPM device (CareFusion Germany 234 GmbH, Hoechberg, Germany). Three attempts were conducted for both MIP and MEP, with the highest value recorded as the achieved result. To assess the change in muscle strength, measurements were conducted on baseline, day 14 and day 35.

All statistical analyses were performed using SAS^®^ version 9.4 (SAS Institute, Cary, NC, USA) as two-sided on a 5% level of significance and all confidence intervals (CIs) were 95% intervals. Change in a parameter from baseline was analysed using a Restricted Maximum Likelihood (REML)-based Mixed Models for Repeated Measures (MMRM) approach. All subjects with post-baseline data were included with their observed data. Subjects without post-baseline values have change from baseline set to 0 at the first post-baseline visit.

The model includes the fixed, categorical effects of treatment (FDI and FCM), strata, day, treatment-by-day interaction, as well as the continuous, fixed covariates of the parameter baseline value and baseline value-by-day interaction. An unstructured covariance matrix was used to model the within-subject errors. The comparisons were the contrasts between FDI and FCM at the day or days in question based on the least squares means for the treatment-by-day interaction effect. The estimated mean differences based on this model were reported with two-sided symmetric 95% CIs and corresponding p-values (Supporting information Table S15-S18). The Kenward-Roger approximation was used to estimate the denominator degrees of freedom.

Estimates were obtained from a mixed model for repeated measures with study, treatment, day and phosphate subgroup as fixed factors; treatment*day, treatment*phosphate subgroup, day*phosphate subgroup and treatment*day*phosphate subgroup as interaction terms; and baseline value as a covariate.

**References**

1.	Wagner, S.A., et al., *Ferric carboxymaltose increases fracture risk in patients and reduces bone formation in mice with iron deficiency anemia.* Blood, 2026. **148**(1): p. 15-30.

2.	Indelicato, E., et al., *Skeletal muscle proteome analysis underpins multifaceted mitochondrial dysfunction in Friedreich's ataxia.* Front Neurosci, 2023. **17**: p. 1289027.

3.	Zur Nedden, S., et al., *Protein kinase N1 deficiency results in upregulation of cerebral energy metabolism and is highly protective in in vivo and in vitro stroke models.* Metabolism, 2024. **161**: p. 156039.

4.	Reinstadler, V., et al., *A validated workflow for drug detection in oral fluid by non-targeted liquid chromatography-tandem mass spectrometry.* Anal Bioanal Chem, 2019. **411**(4): p. 867-876.

5.	Kockmann, T. and C. Panse, *The rawrr R Package: Direct Access to Orbitrap Data and Beyond.* J Proteome Res, 2021. **20**(4): p. 2028-2034.

6.	Witting, M. and J. Rainer, *Bio- and Chemoinformatic Approaches for Metabolomics Data Analysis.* Methods Mol Biol, 2025. **2891**: p. 67-89.

7.	Pang, Z., et al., *Using MetaboAnalyst 5.0 for LC-HRMS spectra processing, multi-omics integration and covariate adjustment of global metabolomics data.* Nat Protoc, 2022. **17**(8): p. 1735-1761.

8.	Dyar, K.A., et al., *Muscle insulin sensitivity and glucose metabolism are controlled by the intrinsic muscle clock.* Mol Metab, 2014. **3**(1): p. 29-41.

9.	Wolf, M., et al., *Effects of Iron Isomaltoside vs Ferric Carboxymaltose on Hypophosphatemia in Iron-Deficiency Anemia: Two Randomized Clinical Trials.* JAMA, 2020. **323**(5): p. 432-443.
